# Disruption of the C/EBPα—miR-182 balance impairs granulocytic differentiation

**DOI:** 10.1038/s41467-017-00032-6

**Published:** 2017-06-29

**Authors:** Alexander Arthur Wurm, Polina Zjablovskaja, Miroslava Kardosova, Dennis Gerloff, Daniela Bräuer-Hartmann, Christiane Katzerke, Jens-Uwe Hartmann, Touati Benoukraf, Stephan Fricke, Nadja Hilger, Anne-Marie Müller, Marius Bill, Sebastian Schwind, Daniel G. Tenen, Dietger Niederwieser, Meritxell Alberich-Jorda, Gerhard Behre

**Affiliations:** 1 Division of Hematology and Oncology, Leipzig University Hospital, Johannisallee 32a, Leipzig, 04103 Germany; 2 Institute of Molecular Genetics, Academy of Sciences of the Czech Republic, Videnska 1083, Prague 4, 142 20 Czech Republic; 3 Cancer Science Institute, National University of Singapore, 14 Medical Drive, Singapore, 117599 Singapore; 4 Fraunhofer Institute for Cell Therapy and Immunology, Perlickstraße 1, Leipzig, 04103 Germany; 5 Harvard Stem Cell Institute, Harvard Medical School, 3 Blackfan Circle, Boston, MA 02115 USA

## Abstract

Transcription factor C/EBPα is a master regulator of myelopoiesis and its inactivation is associated with acute myeloid leukemia. Deregulation of C/EBPα by microRNAs during granulopoiesis or acute myeloid leukemia development has not been studied. Here we show that oncogenic miR-182 is a strong regulator of C/EBPα. Moreover, we identify a regulatory loop between C/EBPα and miR-182. While C/EBPα blocks miR-182 expression by direct promoter binding during myeloid differentiation, enforced expression of miR-182 reduces C/EBPα protein level and impairs granulopoiesis in vitro and in vivo. In addition, miR-182 expression is highly elevated particularly in acute myeloid leukemia patients with C-terminal *CEBPA* mutations, thereby depicting a mechanism by which C/EBPα blocks miR-182 expression. Furthermore, we present miR-182 expression as a prognostic marker in cytogenetically high-risk acute myeloid leukemia patients. Our data demonstrate the importance of a controlled balance between C/EBPα and miR-182 for the maintenance of healthy granulopoiesis.

## Abstract (web summary)

C/EBPα is a critical transcription factor involved in myelopoiesis and its inactivation is associated with acute myeloid leukemia (AML). Here the authors show a negative feedback loop between C/EBPα and miR-182 and identify this miRNA as a marker of high-risk AML.

## Introduction

Acute myeloid leukemia (AML) is a malignant clonal disease of the haematopoietic system resulting in accumulation of leukemic blasts in the bone marrow, the peripheral blood and casually other tissues^1^. AML can be divided into subgroups by morphology, molecular characterization, and prognosis^2^. Frequent single-gene mutations in AML often affect basic myeloid transcription factors, such as C/EBPα, RUNX1, or PU.1, and are thought to be directly connected to AML initiation^3^.

*CEBPA* encodes the myeloid transcription factor C/EBPα, a master regulator of granulopoiesis^4^. Initiated from alternative start codons, two distinct isoforms are translated, the wild-type 42 kDa form and a truncated 30 kDa isoform^5^. *CEBPA* is mutated in ~10% of AML^6^. Two major types of *CEBPA* mutations exist, N-terminal frameshift mutations usually preserving the truncated p30 isoform and affecting the transactivation capacity of C/EBPα, and C-terminal in-frame mutations disrupting the DNA binding and protein–protein interaction of C/EBPα^7^. Inactivation of C/EBPα by other mechanisms, such as promoter hypermethylation or posttranslational modifications, have also been described in patients with AML^8–12^.

MicroRNAs (miRNAs), a class of small non-coding RNAs, are important regulators of normal haematopoiesis and leukemia development^13^. They bind to the 3′ untranslated region (3′UTR) of target messenger RNAs (mRNAs) through an imperfect match, which leads to mRNA destabilization and/or translational inhibition^14^. MiRNAs affect basic cellular functions, such as proliferation, differentiation, and apoptosis^15, 16^, and are involved in various steps of haematopoiesis, including early stem cell maintenance^17^ and myeloid differentiation^18, 19^. On the one hand, we and others have already shown that miRNAs can act as strong oncogenes in AML^20, 21^. On the other hand, we have also shown that miRNAs are common direct targets of C/EBPα during myeloid differentiation and tumor suppressors in AML^22–24^. Although C/EBPα has typically been described as a transcriptional activator^25^, evidence indicates that inactivation of proto-oncogenic target genes is a common and crucial function of C/EBPα^26, 27^. To our knowledge, the importance of C/EBPα-mediated suppression of oncogenic miRNAs in promoting myelopoiesis has not been shown.

Here, we show miR-182 is a downstream target that is negatively regulated by C/EBPα during myeloid differentiation. Furthermore, we demonstrate a feedback mechanism in which C/EBPα is a target of miR-182 in AML. Moreover, high miR-182 expression associates with adverse prognosis in high-risk AML. Altogether, our results suggest that the C/EBPα-miR-182 balance critically modulates granulopoiesis in AML.

## Results

### C/EBPα blocks miR-182 expression

**Fig. 1:**
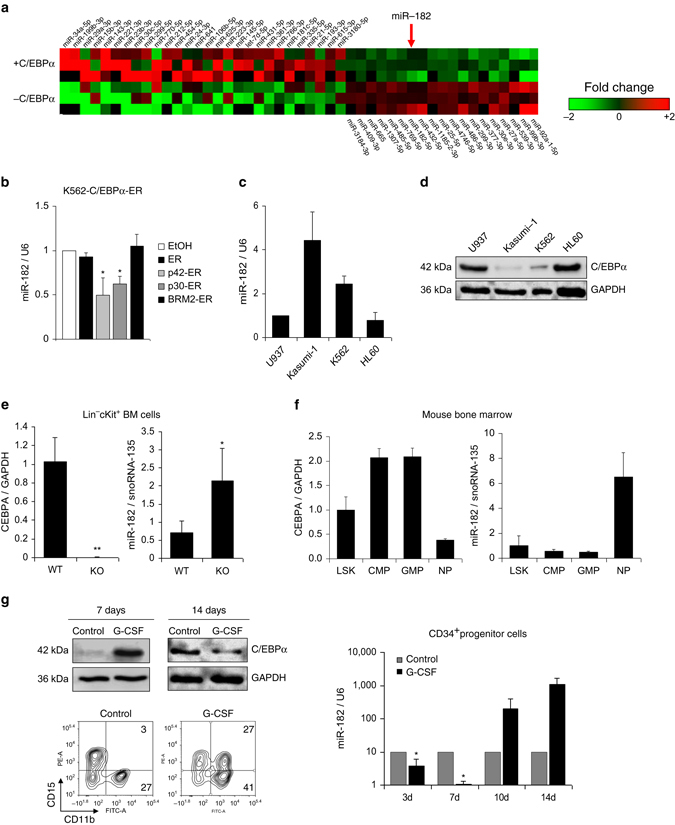
C/EBPα blocks miR-182 expression and both factors correlate inversely. **a** Next Generation Sequencing (NGS) for small RNAs in inducible K562-C/EBPα-ER cells revealed several miRNAs upregulated and downregulated by C/EBPα. MiR-182 is highlighted in bold *(red arrow*). Data summarize three independent experiments, while each colored box marks the relative expression of the indicated miRNA in comparison to the appropriate mean of each experiment. Red represents high fold change and green low fold change. **b** QPCR for miR-182 expression showed downregulation of miR-182 after induction of wild-type C/EBPα-p42 in K562-C/EBPα-ER cells. Induction of N-terminal mutant C/EBPα-p30, but not C-terminal mutant C/EBPα-BRM2 or control ER resulted in reduced miR-182 expression levels. Ethanol served as vehicle control. Cells were collected after treatment with β-estradiol for 6 h. Data represent the mean ± SD of three independent experiments (*p < 0.05). *P* values were calculated using unpaired Student’s *t*-test. **c** and **d** Relative expression levels of miR-182 were analyzed by qPCR **c** and correlate inversely to the protein amount of C/EBPα **d** in indicated leukemic cell lines, as analyzed by western blot. **e** Induced knockout of *CEBPA* by injection of pI:pC in *CEBPA* ^f**/**f^Mx1Cre^+^ mice led to reduced *CEBPA* mRNA levels compared to pI:pC injected *CEBPA* ^f/f f^Mx1Cre^−^ mice and resulted in an upregulation of miR-182 in lineage^−^/c-Kit^+^ myeloid progenitor cells. Expression was measured using qPCR and quantified compared to the mean of all animals. Data represent the mean ± SD of four animals (**p < 0.01). *P* values were calculated using unpaired Student’s *t*-test. **f** Relative expression levels of miR-182 and *CEBPA* in sorted murine bone marrow subpopulations. LSK—Lineage^−^Sca-1^+^cKit^+^ myeloid progenitors, CMP—common myeloid progenitors, GMP—granulocyte-macrophage progenitor, NP—neutrophils. **g** Treatment of human CD34^+^ hematopoietic progenitor cells with G-CSF in vitro led to a rapid decrease of miR-182 expression and correlated inversely to C/EBPα protein levels. C/EBPα protein level was analyzed using western blot (*left side, upper panel*). The stage of differentiation was shown by flow cytometry after 14 days (*left side, lower panel*). MiR-182 expression was measured using qPCR (*right panel*). Normalization for miR-182 expression was done using U6 (human samples) or snoRNA-135 (mouse samples). For *CEBPA* expression normalization, GAPDH was used. Data represent the mean ± SD of three independent experiments (*p < 0.05). *P*-values were calculated using unpaired Student’s *t*-test

In order to identify potential target miRNAs of C/EBPα, we performed next generation sequencing for small RNAs in K562-C/EBPα-ER cells (Supplementary Fig. 1a). After treatment with β-estradiol (E2), C/EBPα is translocated into the nucleus, binds to target promoter regions and effectively induces myeloid differentiation. K562 cells lacking C/EBPα (K562-ER) are unable to trigger those effects (Supplementary Fig. 1b). We identified 28 miRNAs upregulated and 19 miRNAs downregulated by C/EBPα (Fig. 1a, Supplementary Tables 1 and 2). Known C/EBPα target miRNAs miR-34a-5p, miR-29a-3p, miR-30c-5p and miR-223-3p^22–24, 28^ served as positive controls. Within these findings, we detected miR-182 as potential candidate miRNA that is downregulated by C/EBPα (Fig. 1a and Supplementary Table 2). Since it was shown to be oncogenic in several solid tumors^29, 30^ and rarely studied in AML, we focused further investigations on miR-182. We confirmed the C/EBPα-wild-type (p42) dependent effects on miR-182 expression by quantitative real-time PCR (qPCR) in the same model system (Fig. 1b and Supplementary Fig. 1c). Noticeably, N-terminal truncated isoform C/EBPα-p30 was still able to repress miR-182 expression, while C-terminal mutant C/EBPα-BRM2, as well as control ER activation did not affect miR-182 expression (Fig. 1b and Supplementary Fig. 1d–f). Since we hypothesize a direct connection between C/EBPα and miR-182, we compared expression of miR-182 to C/EBPα protein levels in various leukemic cell lines. K562 and Kasumi-1 cells showed high miR-182 expression (Fig. 1c), whereas C/EBPα was not present at protein level (Fig. 1d). In contrast to this, U937 and HL-60 cells exhibited low miR-182 expression and high C/EBPα levels. As an induced knockout of *CEBPA* results in the complete loss of mature neutrophils in *CEBPA* ^f/f^Mx1-Cre^+^ (*CEBPA* KO) mice^31^, we were interested if this leads to an alteration of miR-182 expression in vivo. Here, we demonstrated that miR-182 expression was elevated in lineage-negative/c-kit-positive (lin^−^ckit^+^) myeloid progenitors after knockout of *CEBPA* by injection of pl:pC (Fig. 1e). In agreement with these findings, miR-182- and *CEBPA* mRNA expression correlated inversely in sorted murine bone marrow subpopulations of C57Bl/6 wild-type mice (Fig. 1f) with the lowest levels of miR-182 in common myeloid progenitors (CMP) and granulocytes-macrophage progenitors (GMP) and the highest expression in mature neutrophils (NP). In contrast, *CEBPA* mRNA levels were increased especially in the CMP- and GMP fraction. It has been reported that C/EBPα is a major component during G-CSF induced granulocytic differentiation of human CD34^+^ hematopoietic progenitor cells^32^. Therefore, we analyzed miR-182 expression after treatment of CD34 enriched progenitor cells from human leukapherisates with recombinant G-CSF in vitro. Here, we demonstrated that C/EBPα protein was increased after 7 days of G-CSF treatment and dropped down after 14 days (Fig. 1g, left upper panel). Administration of G-CSF for 14 days is sufficient to receive a majority of premature and mature neutrophils out of isolated CD34^+^ progenitor cells, as shown by flow cytometry analysis for CD15 and CD11b (Fig. 1g, left lower panel). As a consequence, we observed a strong reduction of miR-182 expression upon G-CSF treatment in early stages of differentiation (until day 7) followed by a strong induction of miR-182 in more mature stages of granulopoiesis (Fig. 1g, right panel). Altogether, our data provide strong evidence of a direct inverse connection between C/EBPα and miR-182.

### *MIR182* promoter is regulated by C/EBPα and E2F

**Fig. 2:**
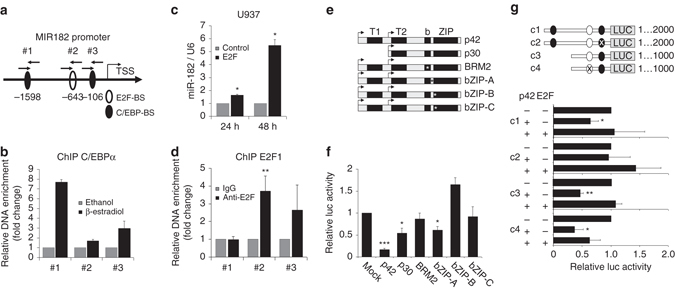
MiR-182 expression is blocked by C/EBPα p42. **a** Schematic overview about the minimal *MIR182* promoter region. **b** ChIP assay in K562-C/EBPα-ER cells revealed a binding of C/EBPα to both distal (#1) and proximal (#3) C/EBP binding sites. Analysis was calculated as a ratio between β-estradiol and ethanol treated cells. Data represent the mean ± SD of two independent experiments. **c** Transient overexpression of E2F1 in U937 cells leads to enhanced miR-182 expression levels at the indicated time points. Expression was measured using qPCR. Data represent the mean ± SD of three independent experiments (*p < 0.05). *P* values were calculated using unpaired Student’s *t*-test. **d** ChIP assay in K562-C/EBPα-ER cells showed a binding of E2F1 predominantly in the region of the predicted E2F binding site (#2). Data represent the mean ± SD of four independent experiments. (**p < 0.01). *P* values were calculated using unpaired Student’s *t*-test. **e** Schematic representation of different C/EBPα mutant forms used for luciferase assay. P42 represents the wild-type, p30 the N-terminal truncated mutant. BRM2 represents a point mutation in the E2F interaction domain at amino acids 294 and 297. The other mutants are insertions in the bZIP domain: bZIP-A (R300-D301 del—ins QN), bZIP-B (dupl. K313) and bZIP-C (dupl. K312). The asterisks mark the region of the point mutation. **f** Luciferase assay with full length *MIR182* minimal promoter showed significantly reduced luciferase activity after C/EBPα-p42, C/EBPα-p30 and bZIP-mutant-A, but not C/EBPα-BRM2, bZIP-mutant-B and bZIP-mutant-C overexpression. **g** Effects of C/EBPα p42 and E2F on luciferase activity can be abolished using specific point mutations in the indicated binding sites. Luciferase activity was measured using different indicated *MIR182* promoter constructs including mutations in the proximal C/EBP site (c2) and the E2F site (c4). Black circles highlight C/EBP binding sites and white circle the E2F site. Co-expression of E2F1 can partially rescue the effect of C/EBPα on promoter luciferase activity. Activity was normalized to pGL3-control. All luciferase experiments were normalized to transfection with equal amounts of pcDNA3.1 empty vector (backbone of all C/EBPα constructs and E2F). Renilla luciferase (pRL) served as internal normalization control. Data represent the mean ± SD of three independent experiments (*p < 0.05; **p < 0.01; ***p < 0.001). *P* values were calculated using unpaired Student’s *t*-test

To answer the question how miR-182 expression is regulated by C/EBPα, we analyzed the minimal promoter region of the *MIR182* gene. Although genomically clustered with two other miRNAs^33^ in the miR-183-96-182 cluster (Supplementary Fig. 2a), it has been shown that *MIR182* exhibits an additional independent promoter region^30^. In consistence with this, we showed that miR-182 does not share the same primary transcript as miR-183 and miR-96 (Supplementary Fig. 2b). Hence, we focused on the 2 kb minimal promoter region of the *MIR182* gene and identified two potential C/EBPα binding sites and one for C/EBPα opponent E2F (Fig. 2a) which are evolutionary highly conserved (Supplementary Fig. 2c) and suit to the current binding motif model for both transcription factors (Supplementary Fig. 2d and e). To test whether C/EBPα directly binds to the MIR182 promoter, we performed chromatin immunoprecipitation assay (ChIP) in K562-C/EBPα-ER cells after induction of C/EBPα. Here, we observed a strong enrichment of DNA fragments amplified with specific primers for both predicted C/EBP binding sites in the *MIR182* promoter region (Primer #1 and #3, Fig. 2b). In addition, the ability of C/EBPα to specifically bind those identified DNA sequences was approved using gel shift assay (Supplementary Fig. 2f). As E2F is a well-known antagonist of C/EBPα and functions in the opposite way in many biological systems^27^, we were interested in the impact of E2F on miR-182 expression. Therefore, we transiently overexpressed E2F1 in U937 cells and analyzed the alterations in miR-182 expression. Here, we noticed a strong increase in miR-182 expression after E2F1 transfection (Fig. 2c). To determine if E2F effects on miR-182 are due to direct promoter binding, we conducted ChIP assay in K562-C/EBPα-ER cells for E2F1. We observed a strong enrichment of the region around the predicted E2F binding site (Primer #2, Fig. 2d). To study the impact of each binding site, we cloned the minimal promoter of the *MIR182* gene into pGL3 luciferase vectors and performed luciferase activity assay in MV4;11 cells. Initially, we used the complete *MIR182* minimal promoter sequence including all three indicated binding sites and performed luciferase assay with different mutant forms of the C/EBPα protein (Fig. 2e). Next to the wild-type p42 and the N-terminal truncated p30 version, we employed different C-terminal mutants of C/EBPα, including the previously published BRM2 mutant^34^ and three different bZIP mutants found in AML patients^35^. We could demonstrate that both exogenous addition of C/EBPα-p42 and C/EBPα-p30, but not C/EBPα-BRM2 decreased luciferase activity, when co-expressed with entire *MIR182* minimal promoter (Fig. 2f). Interestingly, two out of three bZIP mutations (bZIP-B and bZIP-C) failed to block luciferase activity. Only bZIP mutant-A showed the ability to regulate *MIR182* promoter activity even though to a lesser extent than the wild type. In addition, co-expression of E2F1 abolished the effect of C/EBPα-p42 (Fig. 2g). To specify the role of each individual binding site, we inserted either specific point mutations in the proximal C/EBP site (c2) or the E2F site (c4) or used a truncated version of the *MIR182* minimal promoter lacking the distal C/EBP binding site (c3). While mutating the proximal C/EBP site was sufficient to completely abrogate C/EBPα mediated effects, removal of the distal C/EBP site had no effect on luciferase activity. Furthermore, an alteration in the E2F binding motif by mutation prevented E2F1 caused rescue effects (Fig. 2g).

In conclusion, we assume proximal C/EBP binding site (Primer #3) as most important for C/EBPα effects on *MIR182* promoter, and confirmed E2F importance in regulation of miR-182 expression.

### miR-182 defines a subgroup of AML patients and predicts outcome

**Fig. 3:**
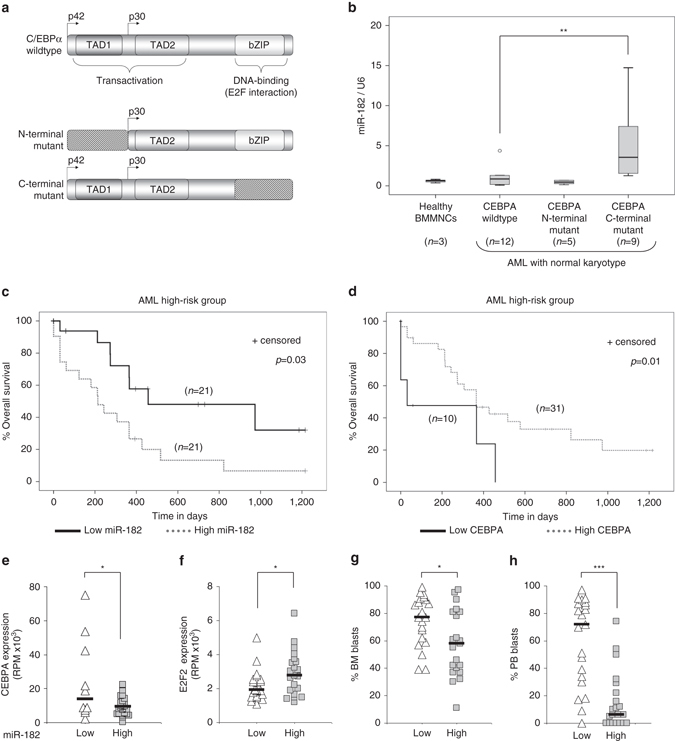
MiR-182 expression defines a subgroup of AML. **a** Schematic representation of different types of C/EBPα found in AML. **b** Relative expression of miR-182 in samples derived from bone marrow mononuclear cells (BMMNCs) of healthy donors (*n* = 3) or normal karyotype AML patients with either *CEBPA* wild-type (*n* = 12), *CEBPA* N-terminal mutation (*n* = 5) or *CEBPA* C-terminal mutation (*n* = 9). MiR-182 expression was elevated only in AML patients carrying a mutation in the C-terminal region of the *CEBPA* gene. Normalization was done using U6 and compared to the mean of all patient samples (** *p*< 0.01). *P* values were calculated using Mann-Whitney-U test. **c** Overall survival of high-risk AML patients according to the median divided miR-182 expression. *P* values were calculated using Log-rank test. **d** Overall survival of according to the *CEBPA* expression. Expression quartiles were created and the group with the lowest *CEBPA* expression (first quartile) was compared to the rest (second, third and fourth quartile). The only existing *CEBPA* mutated patient (ID2952) was excluded from the analysis. *P* values were calculated using Log-rank test. **e** Correlation between miR-182 and *CEBPA* expression in high-risk AML. Groups were divided into four quartiles according to the miR-182 expression. The low group represents the first quartile with the lowest expression levels of miR-182, while the high group combines the rest. Low miR-182 expressing patients showed significantly higher *CEBPA* levels (*n* = 41, *p < 0.05). *P* values were calculated using unpaired Student’s *t*-test. **f** Correlation between miR-182 and *E2F2* expression in high-risk AML. Groups were divided according to the median miR-182 expression. High miR-182 expressing patients showed significantly higher *E2F2* levels. (*n* = 42, *p < 0.05). *P* values were calculated using unpaired Student’s *t*-test. **g** Correlation between miR-182 expression and percentage of bone marrow blasts in the high-risk AML patient set. Groups were divided according to the median miR-182 expression. High miR-182 expressing patients showed significantly lower bone marrow blast counts. (*n* = 42, *p < 0.05). *P*-values were calculated using unpaired Student’s *t*-test. **h** Correlation between miR-182 and percentage of peripheral blood blasts in the high-risk AML patient set. Groups were divided according to the median miR-182 expression. High miR-182 expressing patients showed significantly lower peripheral blood blast counts. (*n* = 42, ***p < 0.001). *P* values were calculated using unpaired Student’s *t*-test

It has been shown that the C-terminal region of the C/EBPα protein is responsible for DNA binding and for E2F interaction^34^ (Fig. 3a). Because of the previous findings, we hypothesized that C/EBPα mediated block of miR-182 is dependent on the C-terminal bZIP domain. To test this, we analyzed the miR-182 expression distribution in primary normal karyotype AML patient samples with either *CEBPA* wild-type, *CEBPA* N-terminal mutation or *CEBPA* C-terminal mutation (including biallelic mutations) (Supplementary Table 3). Here, we demonstrated that AML patients with a C-terminal *CEBPA* mutation showed elevated miR-182 expression levels, while both *CEBPA* wild-type- and *CEBPA* N-terminal mutated AML patients showed no significant changes compared to bone marrow mononuclear cells from healthy donors (Fig. 3b). The same tendency can be found in an AML patient cohort that is provided by The Cancer Genome Atlas^36^ (TCGA) database (Supplementary Fig. 3a and b). Furthermore, miR-182 expression levels were elevated in patients with t(15;17) and t(8;21) translocations by trend (Supplementary Fig. 3c). To address the functional importance of endogenous miR-182 expression in AML, we compared AML patient overall survival to miR-182 expression at diagnosis. Therefore, we used expression data from the TCGA database^36^ and divided all patients into two groups depending on the median expression of miR-182. Here, we observed that high miR-182 expressing patients had a significantly shorter overall survival than low miR-182 expressing patients particularly in the cytogenetically high-risk AML group (Fig. 3c) but not in other subgroups or over all patients (Supplementary Fig. 3d–h). In addition, we examined if *CEBPA* expression is a prognostic factor in the high-risk AML patient set from the TCGA database. Therefore, we compared survival between *CEBPA* low expression group (first quartile) and *CEBPA* high expression group (second to fourth quartile). We could demonstrate that patients with low *CEBPA* expression exhibited a significantly shorter overall survival compared to patients with high *CEBPA* expression (Fig. 3d). Furthermore, we divided all high-risk AML patients into two groups according to the miR-182 expression level and observed a significant inverse correlation to *CEBPA* expression (Fig. 3e), the percentage of bone marrow (BM) blasts (Fig. 3g) and peripheral blood (PB) blasts (Fig. 3h). Especially the percentage of PB blast itself was a strong survival predictor in high-risk AML (Supplementary Fig. 3i). Finally, we found a strong positive correlation between expression values of miR-182 and *E2F2*, a member of the E2F family (Fig. 3f and Supplementary Fig. 3j). Taken together, these data demonstrate the importance of increased miR-182 expression levels in certain subtypes of AML and give strong evidence to the critical domain of C/EBPα for *MIR182* transcriptional regulation.

### C/EBPα is a direct target of miR-182

**Fig. 4:**
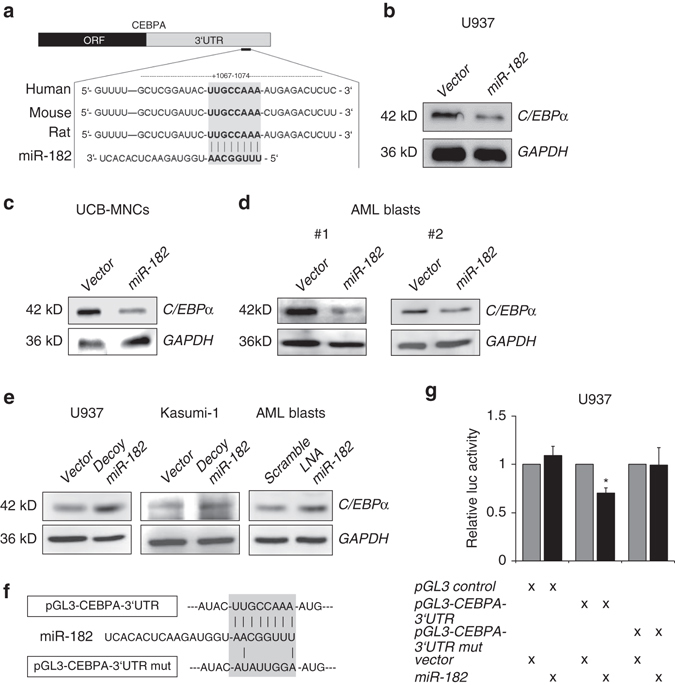
C/EBPα is a direct target of miR-182. **a** Schematic overview about the highly conserved binding site for miR-182 in the 3′-UTR of the *CEBPA* mRNA. **b**–**d** Transient overexpression of human miR-182 led to a reduction of C/EBPα protein amount in **b** U937 cells, umbilical cord blood mononuclear cells (UCB-MNCs) **c** and primary AML-blasts **d** in vitro. **e** A block of miR-182 by either stable infection of U937 or Kasumi-1 cells with decoy-182 vector (*left panels*) or LNA treatment of primary AML blasts in vitro (*right panel*) led to elevated C/EBPα protein levels. **f** Schematic overview about the miR-182 binding site in the luciferase vector and the mutation of the indicated nucleotides. **g** Luciferase reporter and mutagenesis assay. U937 cells were co-transfected with either pGL3-control vector, pGL3-*CEBPA*-3′UTR or pGL3-*CEBPA*-mutant-3′UTR and pcDNA-6.2 empty (*gray bars*) or pcDNA-6.2-miR-182 (*black bars*). An overexpression of miR-182 inhibited the relative luciferase activity only when co-transfected with the pGL3-*CEBPA*-3′UTR. Normalization was done using *Renilla* luciferase construct (pRL). Data represent the mean ± SD of three independent experiments. (*p < 0.05) *P* values were calculated using unpaired Student’s *t*-test

To identify biological important targets of miR-182, we used bioinformatic prediction tool TargetScan (www.targetscan.org). We uncovered a highly conserved putative binding site in the 3′ untranslated region (3′UTR) of the *CEBPA* mRNA (Fig. 4a). To test whether C/EBPα is a direct target of miR-182, we performed transient overexpression of miR-182 and measured subsequent C/EBPα protein levels in diverse in vitro systems. Here, we demonstrated that enforced miR-182 expression led to a rapid decrease of C/EBPα protein levels in U937 cells (Fig. 4b), primary umbilical cord blood mononuclear cells (UCB-MNCs, Fig. 4c), and primary AML patient blasts isolated from AML patient bone marrow (Fig. 4d). In contrast to this, a block of miR-182 expression by either lentiviral expression of a decoy-miR-182 vector in leukemic U937 and Kasumi-1 cells or by treatment of primary AML patient cells with therapeutic dosages of locked nucleic acids (LNAs) against miR-182 resulted in elevated C/EBPα protein levels (Fig. 4e). Finally, a direct binding of miR-182 to the potential binding site in the 3′UTR of the *CEBPA* mRNA was proven by luciferase activity and mutagenesis assay in U937 cells (Figs. 4f and g). Altogether, our results demonstrate that C/EBPα and miR-182 negatively influence each other.

### MiR-182 blocks granulocytic differentiation in vitro

**Fig. 5:**
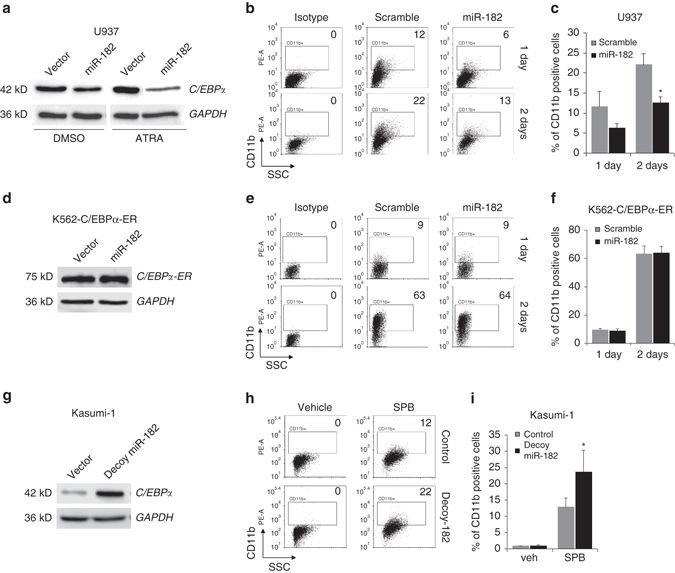
MiR-182 blocks granulocytic differentiation in vitro. **a** Overexpression of miR-182 reduced the amount of C/EBPα protein in both DMSO treated and ATRA treated U937 cells as analyzed by western blot. **b** and **c** Enforced expression of miR-182 led to a reduced percentage of CD11b-positive U937 cells after treatment with ATRA. **b** Representative FACS analysis for myeloid marker CD11b in miR-182 or scramble transfected U937 cells treated for 24 or 48 h with 1 µM ATRA to induce granulocytic differentiation and **c** summary of all experimental repetitions. Data represent the mean ± SD of three independent experiments. (*p < 0.05) *P* values were calculated using unpaired Student’s *t*-test. **d** Overexpression of miR-182 did not affect the amount of C/EBPα-ER fusionprotein in K562-C/EBPα-p42-ER cells as analyzed by western blot. **e** and **f** Enforced expression of miR-182 did not affect the percentage of CD11b-positive K562-C/EBPα-ER cells after treatment with β-estradiol. **e** Representative FACS analysis for myeloid marker CD11b in miR-182 or scramble transfected K562-C/EBPα-ER cells treated for 24 or 48 h with 5 µM β-estradiol to induce granulocytic differentiation and **f** summary of all experimental repetitions. Data represent the mean ± SD of three independent experiments. **g** Block of miR-182 by decoy lentiviral infection increased the amount of C/EBPα protein level in Kasumi-1 cells as analyzed by western blot. **h** and **i** A block of miR-182 accelerates myeloid differentiation of Kasumi-1 cells induced by 1 mM Sodium phenylbutyrate (SPB). **h** Representative FACS analysis for myeloid marker CD11b in decoy-miR-182 or control infected Kasumi-1 cells treated for 72 h with either vehicle control (*left panel*) or 1 mM SPB to induce granulocytic differentiation and **i** summary of all experimental repetitions. Data represent the mean ± SD of four independent experiments. (*p < 0.05) *P* values were calculated using unpaired Student’s *t*-test

It has been shown that C/EBPα is crucial for normal granulocytic differentiation and a loss of C/EBPα is connected to a differentiation block at the stage of early myeloid progenitors^25, 37^. Since miR-182 is able to directly impede C/EBPα protein production, we tested whether miR-182 has a biological impact on myeloid differentiation. Thus, we used all-trans retinoic acid (ATRA) treated U937 cells as model system for human granulocytic differentiation in vitro. As expected, enforced expression of miR-182 in U937 cells treated with ATRA or DMSO control showed reduced C/EBPα protein levels (Fig. 5a). To study the effects of enforced miR-182 expression on myeloid differentiation, we measured cell surface marker CD11b by flow cytometry. Here, we observed that the percentage of CD11b positive cells was remarkably reduced in miR-182 transfected cells compared to control cells after treatment with ATRA (Figs. 5b and c). To prove that these effects are triggered by loss of C/EBPα, we used K562-C/EBPα-ER cells and transfected them with miR-182 or control vector. These cells do not express endogenous C/EBPα^34^, but an artificial C/EBPα-ER fusion protein that is inaccessible for miRNA manipulation due to the lack of a natural 3′UTR (Supplementary Fig. 1a). Here, we could not see an effect of enforced miR-182 expression, neither on the amount of C/EBPα-ER fusionprotein (Fig. 5d) nor on the percentage of CD11b positive cells after treatment with β-estradiol (Fig. 5e and f), indicating that an endogenous C/EBPα expression with a natural 3′UTR is necessary for miR-182 to induce a differentiation block in vitro. Finally, we were interested in the impact of blocking endogenous miR-182 expression on granulocytic differentiation. Therefore, we infected Kasumi-1 cells with a decoy vector against miR-182 or the appropriate control. Reconfirming, this led to enhanced C/EBPα protein levels (Fig. 5g). As this alone was not sufficient to induce myeloid differentiation (Fig. 5h, left panel), we decided to combine miR-182 knockdown with HDAC inhibitor Sodium phenylbutyrate (SPB) treatment, an agent which induces granulocytic differentiation in Kasumi-1 cells^38^. We could show that decoy-miR-182 infected Kasumi-1 cells exhibited accelerated levels of CD11b upon SPB treatment compared to controls (Fig. 5h and i). These findings confirm the strong impact of miR-182 on myeloid differentiation.

### Enforced miR-182 expression blocks G-CSF induced differentiation

**Fig. 6:**
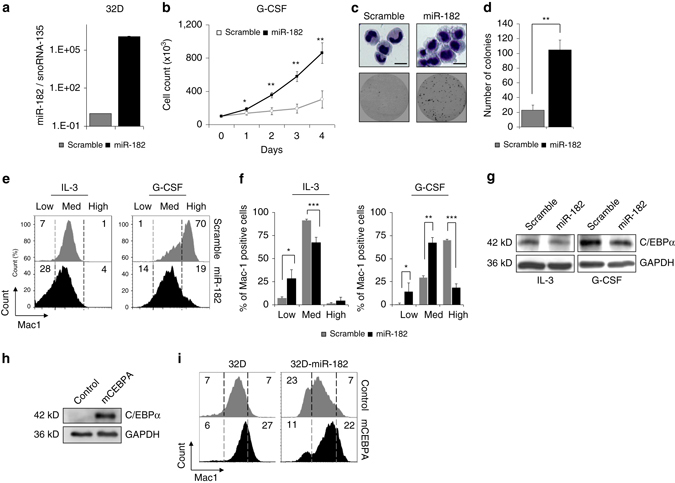
Exogenous C/EBPα abrogates miR-182 mediated effects. **a** MiR-182 levels in 32D cells transduced with either miR-182 or scramble lentivirus. MiR-182 expression was measured by qPCR. **b** MiR-182 expressing 32D cells showed increased cell numbers under G-CSF conditions (*black line*) compared to control scramble transduced cells (*gray line*). 1 × 10^5^ cells were initially seeded and cultured in medium without IL-3 and supplemented with 20 ng/ml G-CSF. Data represent the mean ± SD of three independent experiments. (*p < 0.05; **p < 0.01) *P* values were calculated using unpaired Student’s *t*-test. **c** Morphological analyses by cytospins followed by Wright-Giemsa-staining (*top panel*). MiR-182 expressing 32D cells exhibited less morphological signs of mature granulocytes and remained in an immature stage after treatment with G-CSF for 7 days. Representative plate from a CFU-assay of 32D-scramble or miR-182 expressing cells after 7 days under G-CSF conditions (*bottom panel*). Scale bar 5 µM. **d** Summary of 4 independent CFU-assays demonstrated enhanced colony forming unit ability of miR-182 expressing 32D cells after 7 days under G-CSF conditions. Data represent the mean ± SD. (**p < 0.01) *P*-values were calculated using unpaired Student’s *t*-test. **e** and **f** Stable overexpression of miR-182 led to a reduced percentage of Mac-1 positive 32D cells under both IL-3 and G-CSF conditions. **e** Representative flow cytometry analysis for myeloid marker Mac-1 in miR-182 or scramble expressing 32D cells under IL-3 conditions (*left panel*) or after treatment with 20 ng/ml G-CSF for 7 days (*right panel*) and **f** summary of all experimental repetitions. *Y*-axis indicate the percentage of Mac-1 positive cells. Data represent the mean ± SD of three independent experiments. (*p < 0.05; **p < 0.01; ***p < 0.001) *P*-values were calculated using unpaired Student’s *t*-test. **g** Western blot analysis of 32D cells stably expressing either scramble or miR-182 sequence revealed reduced C/EBPα protein levels under IL-3 conditions or after treatment for 7 days with 20 ng/ml G-CSF. **h** Transduction of 32D cells with exogenous murine *CEBPA* enhances the detectable amount of C/EBPα protein. Analysis was performed using western blot. **i** Exogenous C/EBPα lacking a natural 3′UTR can rescue the miR-182 induced phenotype in 32D cells. Representative flow cytometry analysis for Mac-1 in either parental 32D cells or 32D cells stably expressing miR-182 transduced with control or m*CEBPA*. Analysis was made 10 days after transduction

Next, we were interested if the ability of miR-182 to block myeloid differentiation could be repeated in a G-CSF based model system for granulocytic differentiation. G-CSF is a cytokine that is strongly involved in granulopoiesis in humans and mice in vivo^39, 40^. We stably transduced IL-3-dependent murine 32D cell line with a lentivirus encoding mmu-miR-182 or scrambled control (32D-*MIR182* or 32D-scramble, respectively). After fluorescence activated cell sorting (FACS) for GFP, we measured miR-182 expression by qPCR and observed a strong induction of miR-182 expression in 32D-miR-182 cells (Fig. 6a). An induction of granulocytic differentiation due to a replacement of IL-3 by G-CSF led to a significantly increased cell growth in 32D-miR-182 compared to 32D-scramble cells (Fig. 6b). In addition, 32D-miR-182 cells showed less morphological signs of differentiation under G-CSF conditions (Fig. 6c, upper panel) and an increased number of colonies (Fig. 6c, lower panel and d) compared to 32D-scramble cells in a colony-forming unit assay (CFU-assay). Furthermore, 32D-miR-182 cells showed a reduced surface expression of myeloid marker Mac-1 compared to 32D-scramble cells under both IL-3 and G-CSF conditions (Fig. 6e and f). To explain the biological effects of miR-182 on differentiation of 32D cells, we measured protein levels of C/EBPα by western blot. As expected, C/EBPα protein amount was reduced in miR-182 expressing 32D cells under both IL-3 and G-CSF conditions (Fig. 6g). Moreover, effects of miR-182 on cell surface marker Mac-1 were rescued by co-expression of exogenous C/EBPα lacking a natural 3′UTR (Fig. 6h and i). These findings illustrate the biological importance of the miR-182-C/EBPα-axis for granulocytic differentiation of 32D cells.

### Exogenous miR-182 expression enhances replating capacity

**Fig. 7:**
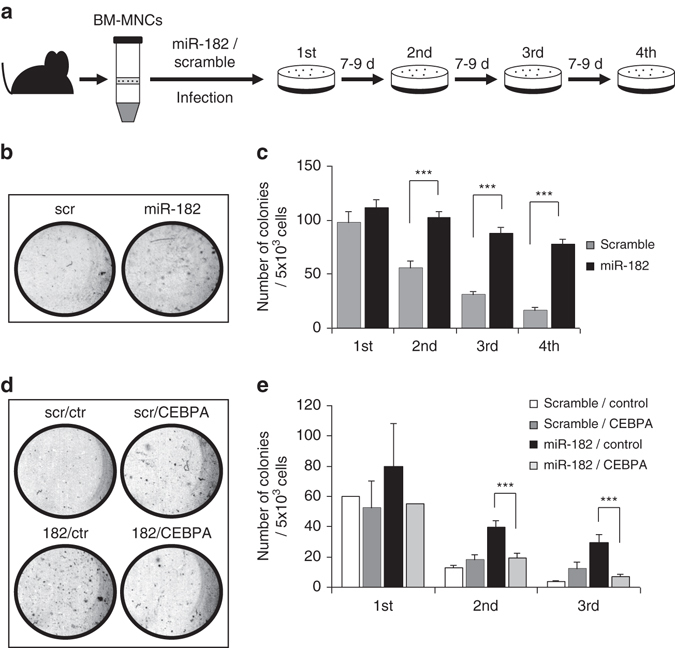
Enforced expression of miR-182 enhances replating capacity. **a** Schematic overview of the experimental strategy. Bone marrow cells were isolated from the tibia and femur and ficolled to obtain the mononuclear cells (BM-MNCs). Cells were infected with virus coding for murine miR-182 or scramble control and sorted for GFP + cells two days after infection. Cells were placed in semisolid medium and replated every 7–9 days. **b** Representative plates of CFU-assay after the fourth round of replating revealed more colonies after enforced miR-182 expression compared to control. **c** Summary of all replating rounds as a result of 4 independent wells of a 12 well plate. MiR-182 expressing mBM-MNCs showed significantly enhanced replating capacity. Data represent the mean ± SD of four independent wells. (***p < 0.001) *P* values were calculated using unpaired Student’s *t*-test. **d** Exogenous C/EBPα lacking a 3′UTR rescues the ability of miR-182 to enhance replating capacity: Representative plates of CFU-assay after third round of replating revealed more colonies after enforced miR-182 expression (182-ctr). This effect was rescued by additional infection with *CEBPA* coding lentiviral vectors (182-*CEBPA*). **e** Summary of all replating rounds as a result of 4 independent wells of a 12 well plate at the second and third replating round. MiR-182-*CEBPA* double expressing mBM-MNCs showed significantly reduced replating capacity compared to miR-182-control expression cells. Data represent the mean ± SD of four independent wells. (***p < 0.001) *P* values were calculated using unpaired Student’s *t*-test. scr = miR-scramble control vector, ctr = *CEBPA* empty control vector

To analyze the function of miR-182 as a potential oncogene, we manipulated isolated bone marrow mononuclear cells from C57Bl/6 mice by lentiviral infection with miR-182 or scramble coding sequences. After sorting for GFP, we plated 5 × 10^3^ cells per well in a semisolid medium supplemented with myeloid specific cytokines, and performed replating assay for four rounds (Fig. 7a). Here, we observed that cells expressing miR-182 showed significantly increased ability to form colonies compared to scramble controls (Fig. 7b and c). As we identified C/EBPα as an important target of miR-182, we were interested if addition of exogenous C/EBPα could rescue the miR-182 mediated effects. Therefore, we infected isolated murine bone marrow cells with both GFP labeled miR-182 and mCherry labeled *CEBPA* lentiviral particles or appropriate controls. GFP^+^mCherry^+^ cells were FACS sorted and plated in 5 × 10^3^ cells per well in a semisolid medium under conditions described above. Here, addition of exogenous C/EBPα led to a significant reduced number of colonies after second and third replating round compared to miR-182 alone infected cells (Figs. 7d and e). These data suggest an oncogenic potential of miR-182 in murine bone marrow cells, which can be rescued by C/EBPα.

### Enforced miR-182 expression impairs granulopoiesis in vivo

**Fig. 8:**
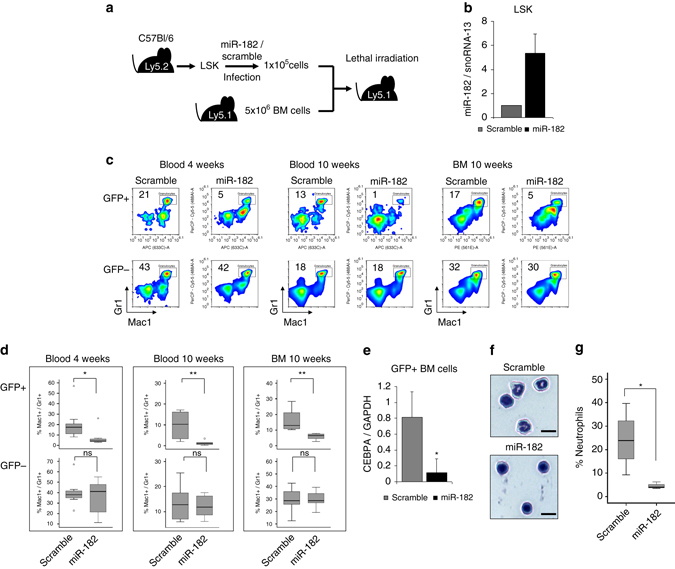
Enforced expression of miR-182 blocks granulocytic differentiation in vivo. **a** Schematic overview of the LSK based transplantation model. **b** MiR-182 levels in GFP^+^ LSK cells transduced with either miR-182 or scramble lentiviral particles. MiR-182 expression was measured by qPCR 48 h after transduction. **c** Representative flow cytometry analysis from blood (4 and 10 weeks) and bone marrow (10 weeks) after transplantation revealed a reduced percentage of Mac1^+^/Gr1^+^ granulocytes in the GFP^+^ fraction of mice transplanted with miR-182 transduced LSK cells compared to mice transplanted with scramble transduced cells. GFP^−^ cells served as controls. **d** Summary of flow cytometry analysis from blood and bone marrow at indicated time points after transplantation. Percentage of mature Mac1^+^/Gr1^+^ granulocytes in the GFP^+^ fraction of mice transplanted with miR-182 transduced LSK cells was significantly reduced. No effect was observed in the GFP^−^ fraction. Bars represent the summary of 3 independent bone marrow transplantations with the indicated total number of animals (4 weeks: scramble *n* = 9 and miR-182 *n* = 8; 10 weeks: scramble *n* = 8 and miR-182 *n* = 6; *p < 0.05; **p < 0.01; ns—not significant). *P* values were calculated using unpaired Student’s *t*-test. **e** MiR-182 expressing mice showed reduced *CEBPA* mRNA levels in the bone marrow. GFP + cells from the bone marrow 10 weeks after transplantation were sorted and analyzed by qPCR for *CEBPA* mRNA expression. Data represent the mean ± SD of four independent animals. (*p < 0.05) *P* values were calculated using unpaired Student’s *t*-test. **f** and **g** Cell morphology analysis from GFP^+^ sorted bone marrow cells 10 weeks after transplantation by Wright-Giemsa-staining showed reduced numbers of mature neutrophils in miR-182 transduced mice. **f** Representative photographs (Scale bar 5 µM) and **g** summary of four mice (scramble) and three mice (miR-182), respectively. (*p < 0.05) *P* values were calculated using unpaired Student’s *t*-test

In order to investigate the oncogenic potential of enforced miR-182 expression in vivo, we performed bone marrow transplantation (BMT) experiments with manipulated lineage^–^ Sca-1^+^ cKit^Hi^ (LSK) cells, a murine bone marrow population enriched for hematopoietic stem and progenitor cells (Fig. 8a). Therefore, purified LSK cells from C57Bl/6 Ly5.2 mice were treated with lentiviral particles coding for either miR-182 or scramble control. We demonstrated that miR-182 manipulated LSK cells showed a strong increase in miR-182 expression in vitro (Fig. 8b). Transduced LSK cells were mixed with 5 × 10^6^ whole bone marrow cells (Ly5.1) and transplanted into lethally irradiated Ly5.1 congenic C57Bl/6 mice. Recipient mice were examined for hematopoietic recovery and lineage development 4 weeks (blood) and 10 weeks (blood and bone marrow) after transplantation. In general, the recovery of Mac1 expressing myeloid cells after transplantation of LSK cells occurs much faster than the recovery of lymphoid cells. This led to a shifted myeloid cell/lymphoid cell ratio 4 weeks after transplantation and was regenerated at 10 weeks post BMT (Supplementary Fig. 4a). Donor manipulated Ly5.2 cells were distinguished from carrier and recipient Ly5.1 cells by flow cytometry for CD45.2, CD45.1, and GFP expression (Supplementary Fig. 4b). First, we gated Ly5.2^+^ and Hoechst^−^ cells (representing the living Ly5.2 population) and divided it into GFP^+^ (miR-182/scramble expressing) and GFP^−^ (non-infected cells) fraction. We observed a strong reduction in the percentage of Mac1^+^/Gr1^+^ mature granulocytes in the GFP^+^ fraction of the miR-182 manipulated group in blood 4 weeks as well as 10 weeks after transplantation (Fig. 8c and d). Furthermore, these effects were also monitored in bone marrow 10 weeks after BMT (Fig. 8c and d), again only in the GFP^+^ fraction of the miR-182 group. There was no significant difference in the percentage of Mac1^+^/Gr1^+^ granulocytes in the GFP^−^ fraction, neither in blood nor bone marrow (Fig. 8c and d). To investigate the effects of enforced miR-182 expression in vivo on its target C/EBPα, we measured *CEBPA* mRNA levels by qPCR in GFP sorted cells from the bone marrow 10 weeks after transplantation. Here, we detected significantly reduced *CEBPA* levels in miR-182 expressing cells (Fig. 8e). Notably, the number of detectable GFP^+^ cells in the blood and bone marrow decreased over time indicating that neither miR-182 nor scramble expressing progenitor cells revealed a proliferation advantage (Supplementary Fig. 4e). To further verify the flow cytometry findings, we performed morphological analysis by cytocentrifugation followed by Wright–Giemsa staining of the previously described sorted GFP^+^ bone marrow cells from recipient mice 10 weeks after BMT (Fig. 8f). We observed a strong reduction of mature granulocytes after enforced miR-182 expression compared to the control group (Fig. 8g). In addition, we were interested if a reduction of mature granulocytes might lead to an increased number of other mature hematopoietic cells. Therefore, we stained blood and bone marrow from recipient mice for monocytic/macrophage and B-cell-specific cell surface markers at different time points and analyzed their distribution using flow cytometry. Here, we did not observe stable and reproducible alterations in the percentage of Mac1^+^/F40-80^+^ monocytes/macrophages (Supplementary Fig. 4c) or B220^+^ B-lymphocytes (Supplementary Fig. 4d) in blood or bone marrow. Taken together, enforced expression of miR-182 blocks specifically granulocytic differentiation in vivo but does not promote monocytic/macrophage or lymphocytic differentiation.

## Discussion

Development of acute myeloid leukemia is connected to deregulation of genes, frequently those coding for transcription factors^3^ or small non-coding RNAs^13^. In the present study, we identified miR-182 as negative target of myeloid transcription factor C/EBPα. As a transcriptional activator^25^, C/EBPα can effectively activate differentiation associated target genes, such as G-CSF receptor or myeloperoxidase^41^. We and others demonstrated that C/EBPα is an activator of tumor suppressive miRNAs miR-30c^22^, miR-223^23^, miR-34a^24^, and miR-29a/b^28^. In contrast, growing evidences suggest that the ability of C/EBPα to negatively regulate target genes by interfering with proto-oncogenic transcription factors is at least as important as its activating function for normal myelopoiesis. C-Myc^42^ and other genes^26, 27^ were described to be blocked by C/EBPα. An activation of C/EBPα in human CD34^+^ hematopoietic progenitors or K562 cells leads to both activation, as well as inactivation of specific groups of target genes^43, 44^. To answer the question how transcriptional activator C/EBPα can inhibit target gene expression, a common mechanism has been presented: one of the major opponent factors of C/EBPα function is cell cycle regulator E2F^45^. Several groups revealed that C/EBPα is able to directly bind to E2F on a target promoter and inhibit its transcriptional activity^27, 42^. Interestingly, we discovered a similar mechanism for C/EBPα during miR-182 expression control. While a direct binding of C/EBPα to the *MIR182* minimal promoter could be proven using ChIP assay, E2F1 seems to be a strong activator of miR-182 expression and thus a functional antagonist of C/EBPα within miR-182 regulation. Notably, we found that activation of N-terminal truncated C/EBPα-p30 mutant still represses miR-182 expression, although to a lesser extent, while different C-terminal *CEBPA* mutants do not. It has been shown that induction of C/EBPα-p30 mutant in mice promotes myeloid commitment and development of leukemia^46^. In contrast to this, incorporation of BRM2 C-terminal *CEBPA* mutant fails to induce lineage commitment in mice^47, 48^ and results in a phenotype comparable to that of *CEBPA* null mice^37^. It has also been shown that C-terminal *CEBPA* mutants completely lose the physical interaction ability to E2F, while N-terminal mutants still bind to E2F^34^. We conclude that the C-terminal domain of the C/EBPα protein and its interaction ability with E2F is crucial for C/EBPα mediated inhibition of miR-182 transcription. In agreement with these findings, we could demonstrate that miR-182 expression is elevated specifically in AML patients with C-terminal *CEBPA* mutations and in AML patients with *t*(15;17) and *t*(8;21) translocations. Both translocation subtypes are connected to reduced C/EBPα protein levels^49, 50^.

Expression of functionally important miRNAs were discussed to predict AML patient outcome^51^. Especially in adverse-risk karyotype AML patients the identification of novel survival predictors might enhance the poor treatment response. In this study, we identified miR-182 expression as a strong prognostic factor in high-risk AML. Unlike proposing it as an independent prognostic factor, we found a strong inverse correlation of miR-182 expression to the expression of *CEBPA* and the percentage of blasts in the peripheral blood and the bone marrow. MiR-182 was reported to be increased in leukemia initiating AML cells^52^ compared to leukemic blasts. Within this work, we also demonstrated that it is heavily expressed in mature myeloid cells. This might explain the inverse connection of miR-182 expression to peripheral blood and bone marrow blasts. In addition, we found a positive correlation between miR-182 and *E2F2* expression in AML patients. E2F2 is a member of the E2F family and associated to cell cycle progression^53^. Altogether, these findings prove the functional relevance of endogenous miR-182 expression in AML.

C/EBPα is inactivated in ~50% of all AML cases^12^. In the present study, we discovered a novel mechanism how C/EBPα can be repressed in AML by specific microRNA interactions. Here, we demonstrated that miR-182 directly blocks C/EBPα during myeloid differentiation and in AML. In contrast to this, inhibiting miR-182 in AML increases C/EBPα protein levels. It has been shown that C/EBPα can be blocked by miR-124^10, 12^ and by miR-690^11^ in several contexts. But to our knowledge, a miRNA based inhibition of C/EBPα during hematopoietic differentiation or during AML development has not been demonstrated.

Oncogenic miRNAs are described to be able to block differentiation, induce proliferation and promote leukemia development^54, 55^. They are also discussed to be potential therapeutic targets in AML^56^. MiR-182 was shown to be strongly oncogenic in gliomas cells^29^ and to promote metastasis of primary sarcomas^30^. In consistence with this, miR-182 was found to be induced by IL-3 and blocked by G-CSF in leukemic cells^57^. It has also been shown that miR-182 is regulated by STAT5 during T-cell proliferation^58^. In the present study, we could clearly prove the oncogenic potential of miR-182 in hematopoietic cells and its ability to impair granulocytic differentiation in vitro and in vivo. Noticeably, the capability of miR-182 to compromise granulocytic differentiation seems to be dependent on its C/EBPα repressing activity, as overexpression of miR-182 in K562-C/EBPα-ER cells (that express an artificial C/EBPα construct lacking a natural 3′UTR)^34^ does not affect the differentiation process after treatment with β-estradiol. Furthermore, addition of exogenous *CEBPA* without a natural 3′UTR can rescue the pre-leukemic phenotype induced by enforced miR-182 in a murine bone marrow replating assay.

Enforced expression of miR-182 in LSK cells leads to a reduced percentage of mature granulocytes in the blood and bone marrow in vivo without developing a leukemia-like disease. A similar phenotype has also been shown for transgenic mice harboring C-terminal *CEBPA* mutant BRM2^47^. As we observed elevated miR-182 expression levels in AML patients with C-terminal *CEBPA* mutations and propose a mechanism, where this mutant fails to block miR-182 expression, both systems seem to be comparable. We were also interested if miR-182 manipulation in vivo would promote lineage commitment to the monocytic/macrophage or lymphoid lineage. Repression of C/EBPα is a crucial event during T-cell development^59^. In agreement with that, miR-182 promotes clonal expansion of T helper lymphocytes^58^. Furthermore, a reduction of C/EBPα level during myeloid differentiation favors monopoiesis over granulopoiesis^60^. But despite sporadic alterations in numbers of monocytes/macrophages, B- and T-lymphocytes with certain tendencies over all analyzed mice, we did not observe a stringent shift to a specific lineage in the blood or bone marrow after enforced expression of miR-182 in LSK cells followed by transplantation in irradiated mice. Rather, it seems that there is a block in the granulocytic lineage with a decision to develop another cell type depending on the cell stage and cell fate at the time point when the lentiviral transduction leads to elevated miR-182 levels. We hypothesize that this process is randomized during each experimental repetition. Alternatively, it could also be that the miR-182 expressing cells remain at the precursor cell stage, do not differentiate to mature cells and undergo apoptosis. Other groups discovered that single microRNA manipulation can trigger the development of leukemia^55^ or a myeloproliferative disease^54^. We did not reveal a leukemia-like phenotype or a hyperproliferative disease after miR-182 manipulation in vivo. But as we exhibit C/EBPα as most important target of miR-182 and even a complete loss of C/EBPα in mice does not lead to a lethal leukemia^31^, our findings are not surprising. Moreover, the current model for acute myeloid leukemia initiation assumes that two genetic alterations are causative for the disease onset. While the first genetic variation typically results in a block of differentiation, the second one is connected to hyperproliferation and/or apoptosis prevention^61^. Therefore, it is obvious that enforced miR-182 expression leads to a block of differentiation by reducing C/EBPα protein levels but is not able to induce leukemia^37^. Thus, a deregulation of miR-182 expression could participate in promoting acute myeloid leukemia when combined to a proliferation or anti-apoptosis connected genetic alteration. But nevertheless, we discovered that a block of miR-182 could enhance the effects of drugs to induce differentiation in leukemic cells, probably by raising C/EBPα protein levels. It might be that blocking microRNAs in combination with differentiation or apoptosis inducing chemicals enhance the treatment success during the cancer therapy in the future.

**Fig. 9:**
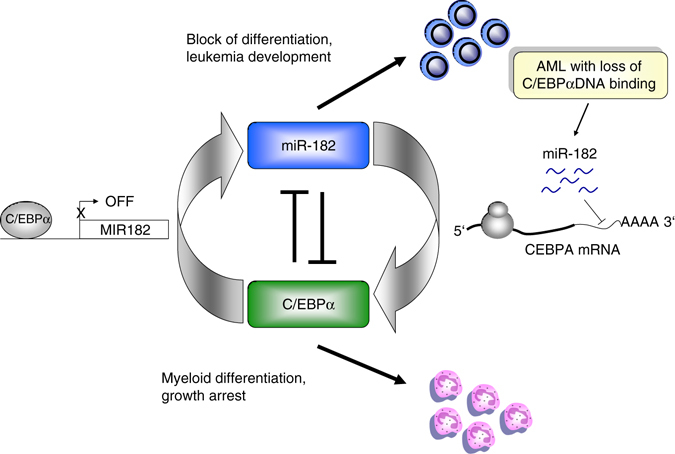
Schematic model of the identified C/EBPα-miR-182 interaction. During myeloid differentiation (*lower panel*), C/EBPα is active, binds to the *MIR182* promoter and reduces miR-182 expression. Elevated miR-182 levels, especially in AML with defective C/EBPα DNA binding capacity, reduce the amount of functional C/EBPα protein by direct binding to the *CEBPA* 3′UTR and block myeloid differentiation (*upper panel*). C/EBPα is a critical transcription factor involved in myelopoiesis and its inactivation is associated with acute myeloid leukemia (AML). Here the authors show a negative feedback loop between C/EBPα and miR-182 and identify this miRNA as a marker of high-risk AML

Taken together, we discovered a novel feedback balance between miR-182 and myeloid transcription factor C/EBPα that is important for a controlled granulopoiesis progress (Fig. 9). A disruption of this balance impairs granulocytic differentiation and seems to be connected to acute myeloid leukemia. Our findings give new insights into the molecular background of the myeloid differentiation cascade and deregulated miRNA-target gene interactions that are connected to acute myeloid leukemia development. The discovery of miR-182 as potential onco-miR in C/EBPα associated AML might also provide novel therapeutic strategies to enhance the treatment response for patients suffering from this disease.

## Methods

### Primary human cell samples

AML patient samples were obtained from Leipzig University Hospital (Leipzig, Germany) and Munich Leukemia Laboratory (Munich, Germany). The study protocols used for AML patient sample collection were approved by Ethical Committee at the Medical Faculty, Leipzig University. All patients provided written informed consent in accordance with the Declaration of Helsinki. All samples were analyzed by cytogenetic and molecular genetic analysis.

Hematopoietic CD34^+^ cells were isolated from leukapherisates using a CD34^+^ MicroBead kit (Miltenyi Biotec), as previously described in ref. ^22^. Umbilical cord blood mononuclear cells (UCB-MNCs) were isolated from fresh umbilical cord blood using human Pancoll (Pan-Biotech) according to the manufacturer’s instructions.

### Cell culture and transfections

K562-C/EBPα-ER (p42, p30 and BRM2) and K562-ER cells were provided by Daniel G. Tenen, Harvard University, Boston, MA, USA and cultured in RPMI-1640 medium (without phenol red) supplemented with 10% charcoal-treated FBS, 1% penicillin/streptomycin/glutamine (PSG) and 1 µg/ml Puromycin. U937 (DSMZ), HL60 (DSMZ) and MV4;11 (DSMZ) cells were maintained in RPMI-1640 medium supplemented with 10% FBS and 1% PSG. Kasumi-1 (DSMZ) cells were cultured in RPMI-1640 medium supplemented with 20% FBS and 1% PSG. 32D (DSMZ) cells were cultured in RPMI-1640 medium supplemented with 10% FBS, 10% WEHI-supernatant and 1% PSG. All cell lines were tested for mycoplasma contamination by the distributor.

Primary human AML patient cells and umbilical cord blood mononuclear cells were cultured in RPMI-1640 medium supplemented with 20% FCS, 1%PSG, 10 ng/ml rh-IL-6, 50 ng/ml rh-SCF and 100 ng/ml rh-Flt3-L. All cytokines were purchased from Immunotools. G-CSF treatment of human CD34 + cells was performed, as previously described in ref. ^22^.

Transfection of U937, UBC-MNCs and AML patient cells was performed using electroporation method with Nucleofactor Kits for cell lines (#VCA-1003 and #VCA-1004, Lonza) according to the manufacturer’s instructions. Transfection efficiency was calculated using flow cytometry for GFP and was >50% in all cell types.

### miRNA expression profiling

MicroRNA expression profiling was performed, as previously described in ref. ^62^. Briefly, 500 ng total RNA extracted using Trizol method was used for small RNA library preparation with the TruSeq Small RNA Sample Prep Kit v2 (#RS-200-0012, Illumina) according to the manufacturer’s instructions. The barcoded libraries were size restricted between 140 and 165 bp, purified and quantified using the Library Quantification Kit-Illumina/Universal (#KK4828, KAPA Biosystems) according to the manufacturer’s instructions. A pool of up to 12 libraries was used for cluster generation per lane. Library DNA at a concentration of 10 pM was clustered using an Illumina cBot according to the SR_Amp_Lin_Block_Hybv8.0 protocol of the manufacturer. Sequencing of 50 bp was performed with an Illumina HighScan-SQ sequencer using version 3 chemistry and the version three flow cell according to the instructions of the manufacturer.

For data analysis, Cutadapt software was used to trim adapter sequences from raw sequences. Alignment to human mature miRNA sequences of miRBase was done from sequences with a length between 15 and 27 bases with bowtie aligner. Quantification of aligned reads was performed using R/Bioconductor programming environment.

### miRNA and mRNA detection by quantitative real-time PCR

Isolation of RNA, reverse transcription and quantitative PCR reaction of miRNAs and mRNA was performed, as previously described in ref. ^22^. RNU6B was used for normalization of human microRNAs, snoRNA-135 for normalization of mouse microRNAs and GAPDH for normalization of mRNAs. All microRNA primers (miR-182, sno-RNA135, RNU6B) were obtained from Life Technologies. Specific mRNA primers (*CEBPA*, *GAPDH*, *E2F1*) were obtained from Biomers and are listed in Supplementary Method section as Supplementary Table 4.

### Reverse transcription and semiquantitative PCR

For reverse transcription (RT), we used RevertAid First Strand cDNA Synthesis Kit (Life Technologies) according to the manufacturer’s instructions. We used oligo-dT primers for the RT reaction. For semiquantitative PCR, we used KAPATaq Extra HotStart Ready Mix with dye (KAPA Biosystems) according to the manufacturer’s instructions.

### ChIP assay

ChIP assay for C/EBPα was performed using ChIP-IT express Kit (# 53008, Active Motif) according to the manufacturer’s instruction. ChIP assay for E2F1 was performed using Magna ChIP A/G Kit (# 17-10085, Millipore) according to the manufacturer’s instruction. For each approach, 2 × 10^7^ K562-C/EBPα-ER cells were treated with β-estradiol or ethanol for 4 h. Sonication was performed with 25% amplitude (1 s pulse followed by 1.5 s pause for 6 min). Anti-C/EBPα antibody (sc-61×) was purchased from Santa-Cruz. Anti-E2F1 antibody (17-10061) was purchased from Millipore. All primers are included in Supplementary Table 4.

### Western blot

Western blots were performed as previously described in ref. ^22^. The following antibodies were used: rabbit mAb Anti-C/EBPα (EP709Y, Abcam), rabbit mAb Anti-C/EBPα (EP708Y, Abcam), rabbit pAb Anti-GAPDH (sc-25778, Santa Cruz Biotech.), rabbit mAb Anti-GAPDH (14C10, CST). Donkey-anti-rabbit-HRP (NA934V, GE-Healthcare) or goat-anti-rabbit (sc-2004, Santa Cruz Biotech.) served as secondary antibodies. For chemiluminescence detection, we used Western Blot Ultra or Western Blot Premium Substrate (Licor) and analyzed the membranes using C-Digit Chemiluminescent Western Blot Scanner (Licor). Evaluation of data were performed using ImageJ software (NIH). Full western blot gel scans are included as Supplementary Figs. 5–8.

### Luciferase assay

Luciferase assay was performed to prove the direct binding of miR-182 to the putative binding site in the 3′UTR of the *CEBPA* mRNA. A ~320 bp fragment surrounding the potential miR-182 binding site was XbaI cloned into the pGL3-control vector. U937 cells were transiently transfected with 0.7 µg pGL3-control, pGL3-*CEBPA*-3-UTR or pGL3-*CEBPA*-3-UTR-mut reporter construct together with either 2 µg pcDNA6.2-miR-182 or pCDNA6.2-control vector. For normalization, 0.1 µg pRL vector (renilla luciferase) was co-transfected.

For promoter luciferase assay, a 1931 bp fragment starting from the transcriptional start site of the *MIR182* gene (c1) or a 1020 bp truncated version lacking the distal C/EBP site (c3) were cloned XhoI/HindIII into pGL3 control vector. For insertion of specific point mutations in either the proximal C/EBP site (c2) or the E2F site (c4), we used QuickChange II XL mutagenesis Kit (#200521, Stratagene).

MV4;11 cells were transiently transfected with 100 ng promoter-pGL3 vector and either mock, 200 ng pcDNA3.1-C/EBPα (p42, p30, or BRM2) or in combination with 200 ng pcDNA3.1-E2F1. All experiments were normalized to the same amount of pcDNA3.1 empty vector. For internal control, 50 ng pRL vector were co-transfected.

All transfections were performed using Lipofectamine LTX (Life Tech.) according to the manufacturer’s instructions. Firefly and renilla luciferase activity was measured 24 h after transfection using Dual-Luciferase Reporter Assay System (Promega) and calculated as ratio between luciferase and renilla activity.

### Gel shift assay

Gel shift assay was performed using LightShift Chemiluminescent EMSA Kit (#20148, Thermo) according to the manufacturer’s instructions. Briefly, 293 T cells were transfected with pcDNA3-C/EBPα-p42 or control. 5 × 10^6^ cells were used to isolate nuclear extracts by NER-PER extraction Kit (#78833, Thermo). DNA-protein binding reaction was performed with 10 µg nuclear extracts with either Biotin-labeled DNA oligo alone or in combination with an unlabeled competitor oligo with the same sequence. Unspecific binding was reduced by addition of Poly (dI-dC). Shift was assigned by incubation of the membrane with Streptavidin-HRP conjugate followed by chemiluminescence detection. Oligo sequences are listed in Supplementary Table 4.

### Cell differentiation, cell count and CFU-assay

To induce granulocytic differentiation in U937 cells, 1 µM all-trans retinoic acid (ATRA, Sigma) dissolved in DMSO was added to the cells. K562-C/EBPα-ER cells were treated with 5 µM β-estradiol (Sigma) dissolved in ethanol. Differentiation status was analyzed using CD11b detection by flow cytometry. In addition, 32D cells were washed twice in phosphate-buffered saline (PBS) and cultured in medium supplemented with 20 ng/ml G-CSF and without WEHI supernatant. Morphological analyses were performed using Wright–Giemsa stained cytospins. For cell count analyses optical density (OD) was measured at 600 nm. For cell number calculation, a standard curve was prepared in advance. Differentiation in Kasumi-1 cells was induced by adding 1 mM Sodium phenylbutyrate (Sigma). Colony-forming unit (CFU) assays were done using 3 × 10^3^ 32D cells per well mixed in MethoCult M3231 (Stemcell Tech.) methylcellulose according to the manufacturer’s instructions. For differentiation, 20 ng/ml rm-G-CSF was added to the methylcellulose. For CFU-replating assay, BM-MNCs were isolated from the bone marrow of C57Bl/6 mice and separated using Pancoll (PAN Biotech), infected with either scramble or miR-182 coding vector and sorted 3 days after transduction for GFP. Immediately after sorting, 5 × 10^3^ cells were seeded per well in MethoCult M3231 (Stemcell Tech.) supplemented with 10 ng/ml rm-GM-CSF, 10 ng/ml rh-IL-6, 10 ng/ml rm-IL-3 and 50 ng/ml rm-SCF. Replating was performed every 7–9 days and colonies were counted every plating round. All cytokines were purchased from Immunotools.

### DNA constructs and cloning

Constructs for C/EBPα-p42, p30, BRM2 and for E2F1 were previously described^7, 23, 47^. Production of C/EBPα bZIP mutants was done using QuikChange II XL Site-Directed Mutagenesis kit (#200521, Stratagene) with the following mutations: bZIP-A (del 899-901 ins AAA—R300-D301 del ins QN), bZIP-B (937-939 dupl—K313 dupl), bZIP-C (934-936 dupl—K312 dupl). AB.pCCL.sin.cPPT.U6.miR-182-Decoy.hPGK.GFP.WPRE (decoy-miR-182) was a gift from Brian Brown (Addgene #46596) and previously published^63^. Vector pRRLSIN.cPPT.PGK-GFP.WPRE (Addgene plasmid # 12252) was a gift from Didier Trono and served as control. Locked-nucleic acids (LNAs) for miR-182 were obtained from Exiqon. Production of murine *CEBPA* lentiviral vector was done by replacing U6 promoter of pSicoR-Ef1a-mCh-Puro by MSCV promoter and cloning ORF of the mouse *CEBPA* gene into BamHI/NotI restricted vector. Previously published pSicoR-Ef1a-mCh-Puro^64^ was a gift from Bruce Conklin (Addgene plasmid # 31845).

Cloning of human pre-miR-182 into pcDNA6.2-GW-emGFP-miR vector was done using Block-iT Pol II miR RNAi Expression Vector Kit (K4935-00, Thermo) according to the manufacturer’s instructions with the following primers: forward 5′-TGCTGTTTGGCAATGGTAGAACTCACACTGGTGAGGTAACAGGATCCGGTGGTTCTAGACTTGCCAACTA-3′ and reverse 5′-CCTGTAGTTGGCAAGTCTAGAACCACCGGATCCTGTTACCTCACCAGTGTGAGTTCTACCATTGCCAAAC-3′.

For Luciferase Assay, a ~320 bp part of the *CEBPA*-wild-type 3′UTR was cloned into XbaI restricted pGL3-control vector (Promega). For *CEBPA*-3′UTR amplification from U937 cDNA, following primers were used: forward 5′- TCTAGAAAGCACGATCAGTCCATCCC-3′, reverse 5′-TCTAGAAGCTGAGGGCAAAGGAGAAC-3′. For mutagenesis of the miR-182 binding site, we used QuikChange II XL Site-Directed Mutagenesis kit (#200521, Stratagene) according to the manufacturer’s instructions and the following primers: forward 5′- CCCCAGCTGCCGACGGAGAGTC TCATTCCAATATGTATCCGAGC AAAACCAAAACAAAACA-3′ and reverse 5′- TGTTTTGTTTTGGTTTTGCTC GGATACATATTGGAATGAGACTCTC CGTCGGCAGCTGGGG-3′. This leads to a t193a_g195a_c196t_c197t_a198g_a199g nucleotide exchange of the *CEBPA* wild-type 3′UTR with the resulting mutated miR-182 binding site ATATTGGA (instead of wild-type TTGCCAAA). Primers for cloning different *MIR182* promoter constructs into pGL3 control are listed in Supplementary Table 4. For insertion of mutations in the binding sites, we converted the proximal C/EBP binding site (site 3) from TGCGACAAG to GCCGAGGGA and the E2F binding site (site 2) from AATGGCGCTGGG to GCTAAAATTGGG.

### Flow cytometry and fluorescence activated cell sorting

For flow cytometry analysis, 1 × 10^6^ cells were washed with PBS and stained for 20 min at 4 °C with specific antibodies, which are listed in the supplement. Subsequently, cells were washed in PBS and measured with BD LSR II cytometer using CellQuest software (BD Biosciences). Final evaluation of data was done using NovoExpress 1.0.2 (ACEA Biosciences). Sorting of GFP^+^ cells and murine bone marrow subpopulations was performed, as previously described^20^. All antibodies are listed in Supplementary Table 5.

### Isolation of bone marrow subpopulations and LSK cell culture

Isolation of LSKs, CMPs, GMPs, and granulocytes was done, as previously descrivbed^22^. Briefly, mouse bone marrow cells were either lineage (CD5, CD45R (B220), CD11b, Gr-1 (Ly-6G/C), 7-4 and Ter-119) depleted or enriched using Lineage Depletion Kit (Miltenyi #130-090-858). Subsequently, cells were stained with specific antibodies and FACS sorted for indicated subpopulations. LSK cells were gated as Lin^-^Sca-1^+^cKit^hi^. CMPs were gated as Lin^−^c-kit^+^Sca1-CD34^+^FcRgII/III^lo^. GMPs were gated as Lin^−^c-kit^+^Sca1^−^CD34^+^FcRgII/III^hi^. Granulocytes were gated as Lin^+^Mac1^+^Gr1^+^. All antibodies are listed in the supplement. Culture of LSK cells was performed, as previously described^27^ in StemSpan SFEM medium (Stemcell Technologies) supplemented with 10 ng/ml mIL-3, 20 ng/ml hIL-6, 100 ng/ml mSCF, 50 ng/ml mTPO, 100 ng/ml mFlt-3 ligand. All cytokines were purchased from Immunotools. All antibodies are listed in Supplementary Table 4.

### Lentiviral transduction

Lentiviral vectors for mmu-miR-182 (MMIR-182-PA-1) and mmu-scramble (MMIR-000-PA-1) were purchased from System Biosciences. Pseudo-viral particles were produced according to the manufacturer’s instructions. 32D cells were infected with 5 multiplicity of infection (MOI) for 24 h, washed two times with PBS and selected with 5 µg/ml Puromycin another 48 h later. Purity of transduced cells was calculated using flow cytometry for GFP expression and was about 100% 7 days after Puromycin supplementation. Stable 32D-GFP-miR-182 (and control) cells were maintained in 32D cell medium supplemented with 1 µg/ml Puromycin. Mouse LSK cells and mBM-MNCs were infected with at least 20 MOIs for 6 h, washed twice with PBS and cultured for another 18 h before transplantation or sorted for GFP and/or mCherry 3 days after infection, respectively.

### Induced *CEBPA* knock out and mouse bone marrow transplantation

Induction of *CEBPA* knock out in vivo and mouse bone marrow transplantation experiments were performed, as previously described^27^. Briefly, *CEBPA* excision in *CEBPA* ^f/f^ Mx1Cre^+^ mice was induced by injection of 500 µg/mouse poly I:C for three times every second day. Cells were collected 11 days after first injection.

For bone marrow transplantation experiments, 1 × 10^5^ pseudoviral particle treated LSK cells from C57Bl/6-Ly5.2 mice were mixed with 5 × 10^6^ total bone marrow cells from C57Bl/6-Ly5.1 mice and transplanted into lethally irradiated C57Bl/6-Ly5.1 randomly grouped mice by retroorbital injection. Peripheral blood was obtained from facial vein 4 weeks after transplantation. Mice were sacrificed 10 weeks after transplantation and peripheral blood and bone marrow was isolated. All mice that showed no proper engraftment (Ly5.2 ≤ 0.5%), did not express GFP or showed general abnormalities in the GFP^-^ fraction were removed from final consideration. All mice experiments were approved by the AnimalCare and Use Committee of the Institute of Molecular Genetics and were in agreement with local legal requirements and ethical guidelines.

### Statistical analyses

We used the Student’s *t-*test to determine the statistical significance of experimental results. A *P* value of 0.05 or less was considered significant. The results were represented as the mean ± SD from at least three independent experiments. For statistical analyses of primary AML patient data, we used Mann-Whitney-U test. A *P* value of 0.05 or less was considered significant.

### Data availability

All data are available from the authors. Data from small RNA deep sequencing have been deposited in the Gene Expression Omnibus (GEO) with the Accession Number GSE72634 and are publicly available.

## Electronic supplementary material

Supplementary Information
