# Supplementary material for: Disruption of the C/EBPα—miR-182 balance impairs granulocytic differentiation
Source: Nat Commun. 2017 Jun 29;8:46. doi: 10.1038/s41467-017-00032-6 (PMC5491528; doi:10.1038/s41467-017-00032-6)
Supplement: Supplementary file 1 — Supplementary Information [file 41467_2017_32_MOESM1_ESM.pdf]

File Name: Supplementary Information
Description: Supplementary Figures, Supplementary Tables, Supplementary Methods and Supplementary References.

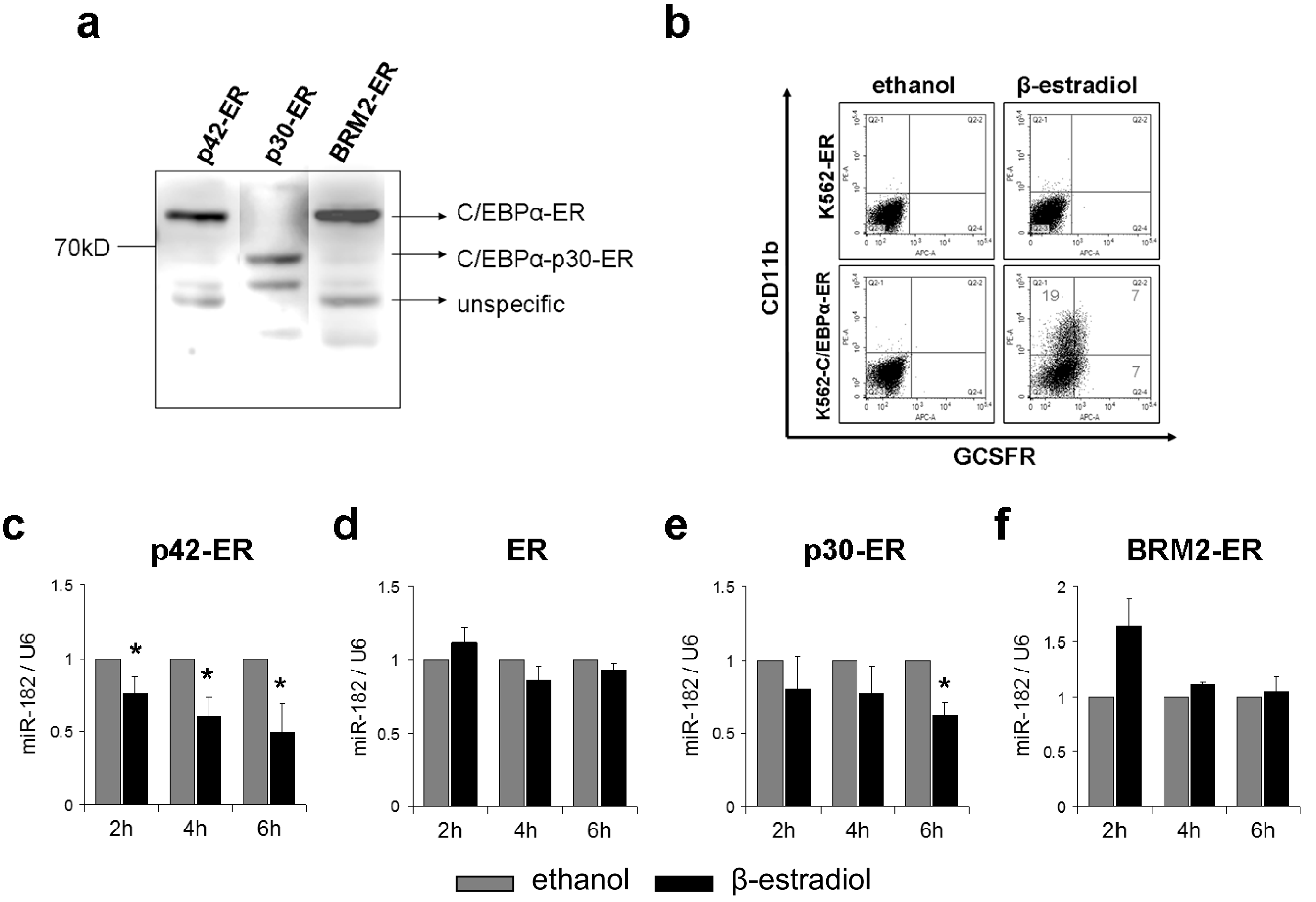

Supplementary Figure 1.

(a) Western blot with anti-C/EBPα-antibody in K562-C/EBPα-p42-ER, K562-C/EBPα-p30-ER and K562-C/EBPα-BRM2-ER cells, respectively. The C/EBPα-ER fusion protein (including the BRM2 mutant) can be detected at approximately 80 kD and the truncated C/EBPα-p30-ER mutant at 65 kD confirming previously published data[1]. **(b)** Flow cytometry analysis for CD11b and G-CSFR revealed that treatment of K562-C/EBPα-ER cells with 5µM β-estradiol rapidly induces granulocytic differentiation. Measurement was done after treatment for 24 hours. K562-ER cells are unable to differentiate under these conditions. Ethanol served as vehicle control. **(c-f)** qPCR analyses of K562-C/EBPα-p42-ER (c), K562-ER (d), K562-C/EBPα-p30-ER (e) and K562-C/EBPα-BRM2-ER (f) treated with 5µM β-estradiol for indicated time points. Y-axes indicate miR-182 expression relative to U6. Data represent the mean ± SD of three independent experiments (*$p < 0.05$). P values were calculated using unpaired Student's t-test.

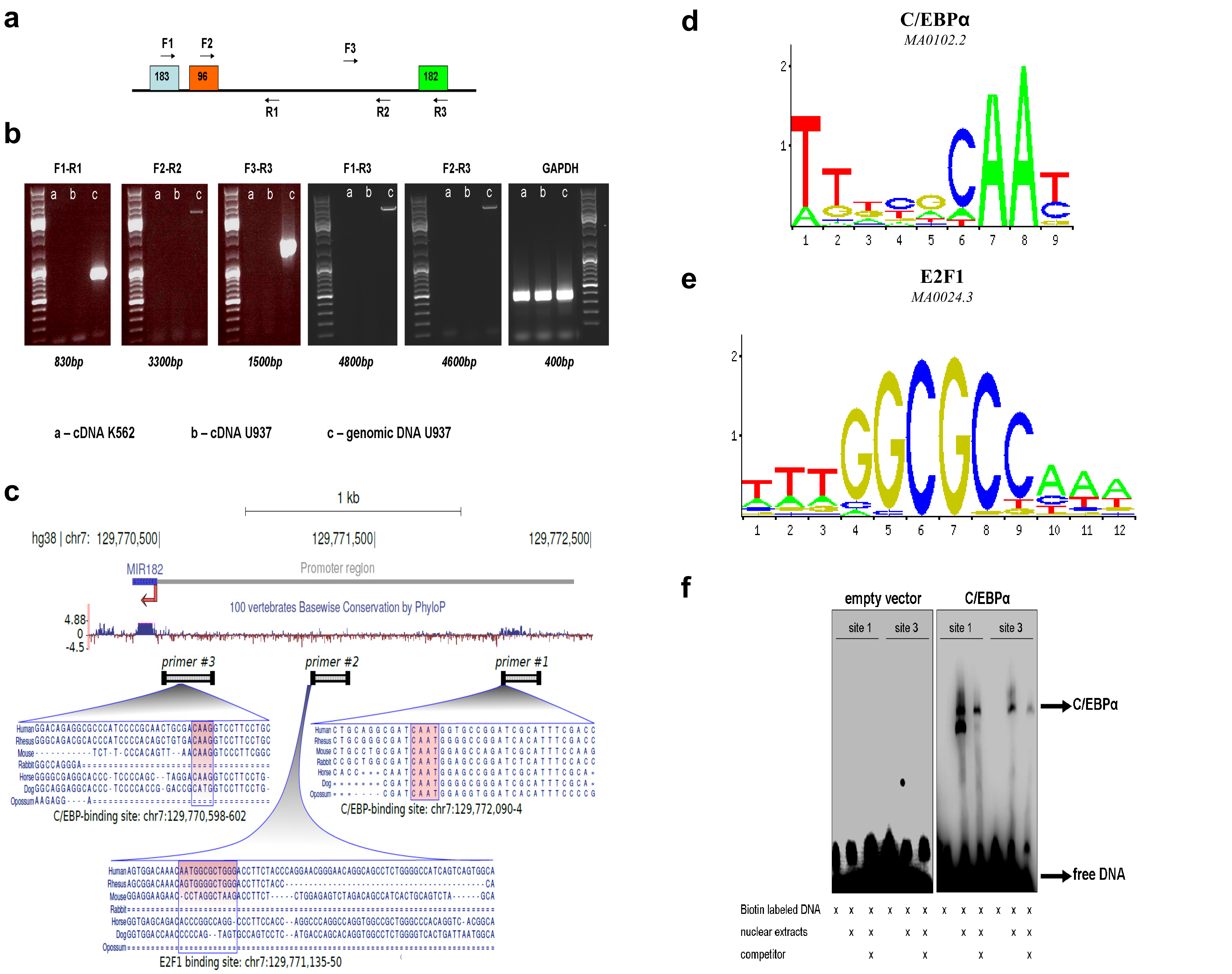

Supplementary Figure 2.
(a) Schematic overview representing the genomic organization of the miR-183-96-182 cluster and the position of the specifically created primers (F1 to R3). The distance between *MIR-96* gene and *MIR-182* gene is more than 4000bp. **(b)** Semi-quantitative PCR to analyze the primary transcript of miR-183, miR-96 and miR-182. While all tested primer pairings showed a specific band in the genomic DNA of U937 cells (positive control, lane c), we could not observe a shared primary transcript of miR-183 together with miR-182 or miR-96 together with miR-182, respectively. Neither cDNA from K562 (lane a) nor from U937 cells (lane b) yielded a specific band after PCR reaction. GAPDH served as control. **(c)** Overview about the MIR-182 minimal promoter (~2000 bp upstream from the transcriptional start site). Highlighted are the identified important transcription factor binding sites and the regions for the ChIP primers used in figure 2. Binding sites for both C/EBPα (primer 1 and primer 3) as well as for E2F1 (primer 2) are well conserved along the species. **(d)** Binding motif for C/EBP transcription factor according to the JASPAR database (http://jaspar.genereg.net). Model number MA0102.2 matches with both identified regions in the *MIR182* minimal promoter.**(e)** Binding motif for E2F1 transcription factor according to the JASPAR database. Model number MA0024.3 matches with the identified region in the *MIR182* minimal promoter. **(f)** C/EBPα binds to both predicted binding sites of the *MIR182* promoter. Gel shift assay with nuclear extracts from 293T cells transfected with either pcDNA3 empty vector (left site) or pcDNA3-C/EBPα-p42 (right site) was performed. Biotin-labeled ds DNA oligos representing the indicated C/EBP binding sites in the *MIR182* promoter were used to illustrate the interaction between C/EBPα and the specific DNA sequence. To prove specificity of the interaction, unlabeled DNA oligos with same sequence were used as competitors and remarkably reduced the amount of shifted DNA. Visualization of the Biotin-labeled DNA was conducted using stabilized Streptavidin-HRP conjugates followed by incubation with chemiluminescent substrate.

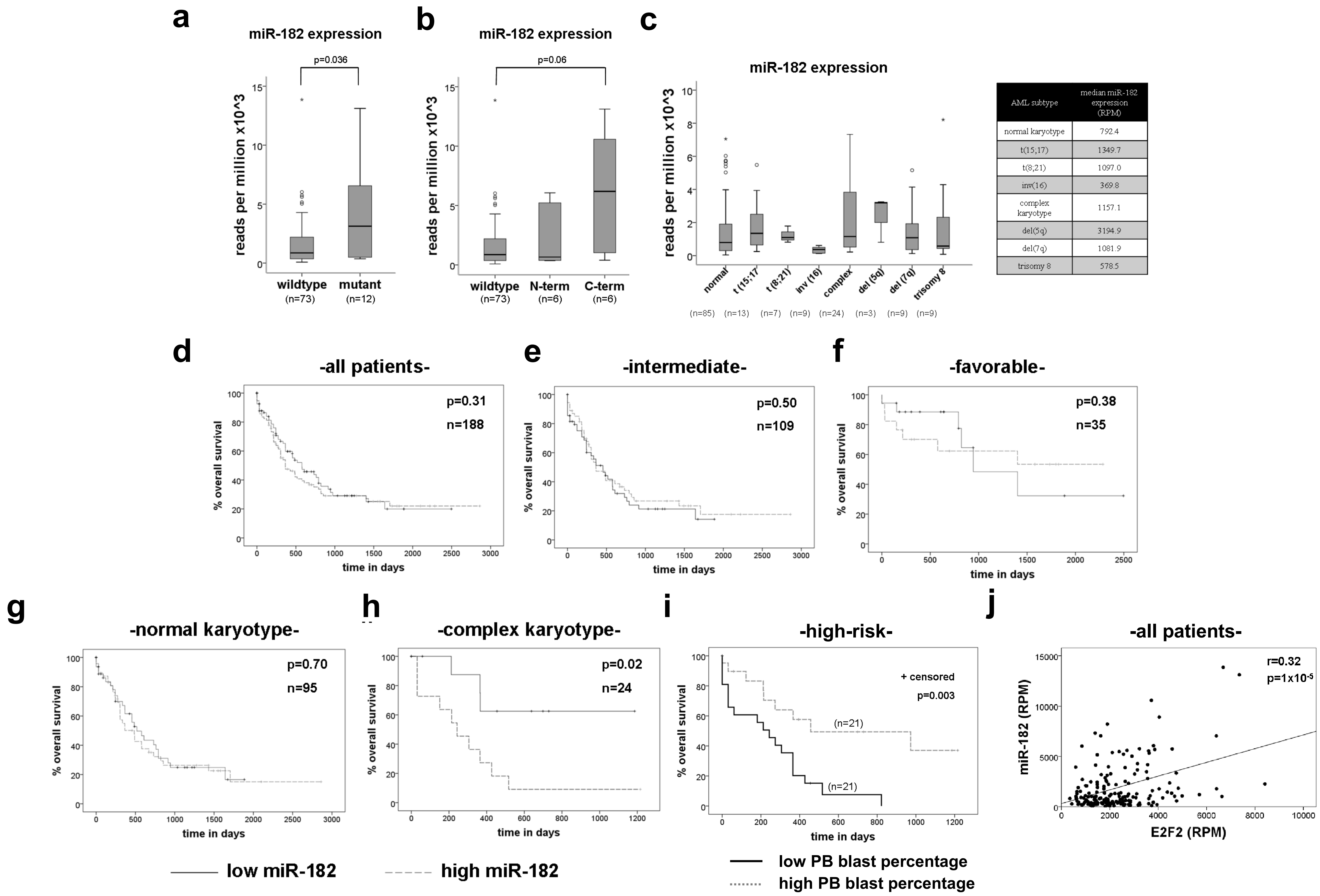

AML subtype	median miR-182 expression (RPM)
normal karyotype	792.4
t(15;17)	1349.7
t(8;21)	1097.0
inv(16)	369.8
complex karyotype	1157.1
del(5q)	3194.9
del(7q)	1081.9
trisomy 8	578.5

Supplementary Figure 3.

(a) MiR-182 expression analyses in AML patients from TCGA database[2] according to the mutation status of *CEBPA*. Grouped patients with any *CEBPA* mutation showed significantly elevated miR-182 expression levels compared to wild-type *CEBPA* patients. Only FAB M1 and FAB M2 patients were considered in both groups. For calculation of significance levels (p=0.036), outliers were not included. P values were calculated using unpaired one-tailed Student's t-test **(b)** Expression levels of miR-182 in N-terminal and C-terminal (including biallelic) subdivided *CEBPA* mutated patients from the TCGA database[2] showed the same tendency like the patients from Figure 3b with the highest levels in C-terminal mutated patients. **(c)** MiR-182 expression levels in AML patients from the TCGA database[2] according to the molecular and cytogenetic subgroup. Patients with t(15;17) and t(8;21) translocations showed higher miR-182 expression levels (median 1349.7 and 1097.0, respectively) compared to normal karyotype patients (median 792.4) by trend. *CEBPA* mutated normal karyotype patients were not included in this consideration. Expression of miR-182 was calculated as sequencing reads per million (RPM). **(d-h)** AML patient overall survival divided into miR-182 high and low expressing subgroups according to miR-182 expression measured at diagnosis via RNA-sequencing and provided by the TCGA database2. Median expression was used to divide between high and low miR-182 group. A significant difference and a survival prediction could only be seen in the complex karyotype group of patients (h, p=0.02) **(i)** Overall survival of AML patients of the cytogenetically high-risk group according to the peripheral blood (PB) blast count. Median of PB blast count was used to divide into low and high (n=42, p=0.003). Data were obtained from the TCGA Research Network.**(j)** Positive correlation between the expression levels of miR-182 (y-axis) and E2F2 (x-axis) over all AML patients measured at diagnosis via RNA-sequencing and provided by the TCGA database[2]. The correlation coefficient r is 0.32 and the p-value 0.00001.

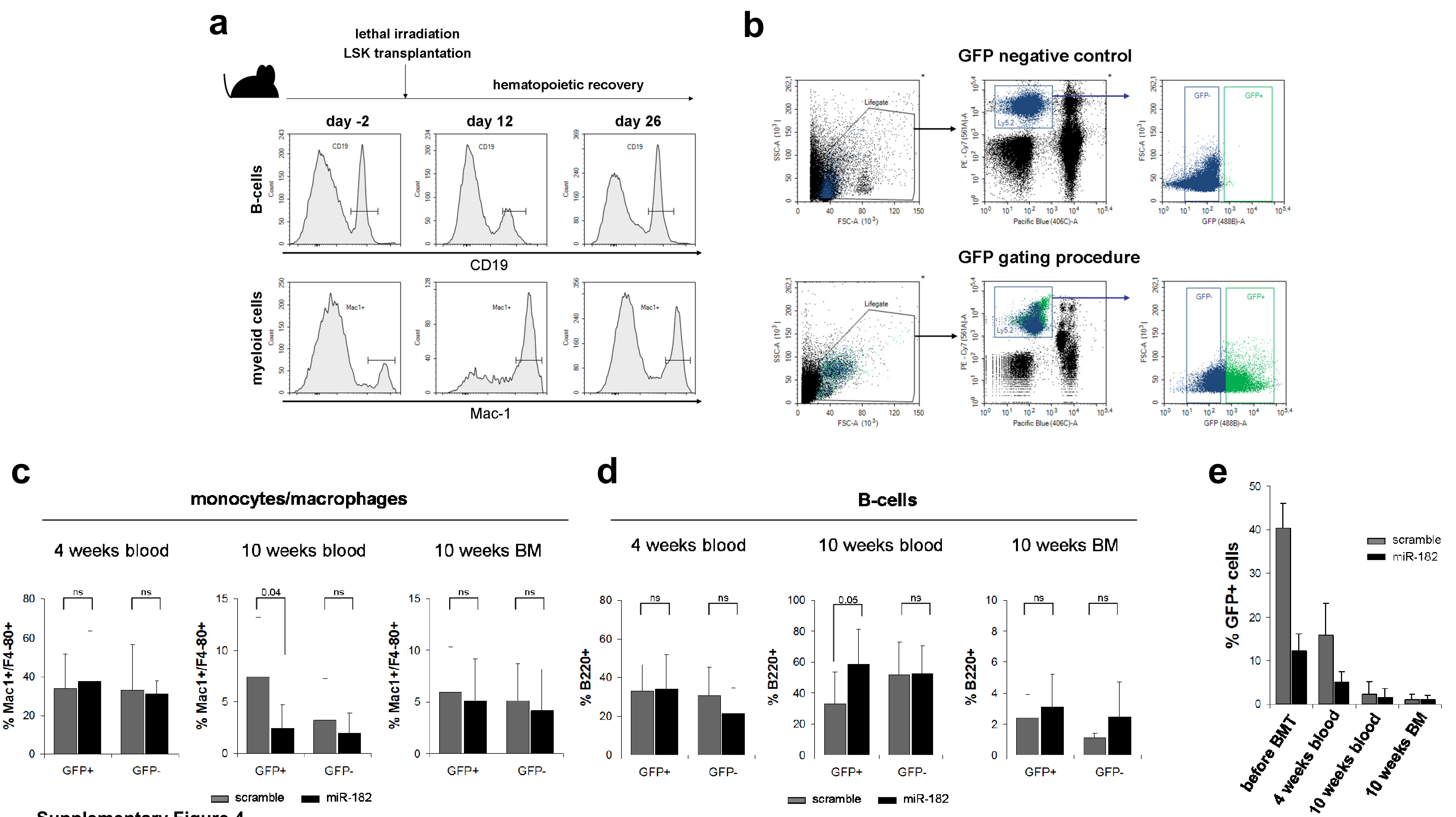

Supplementary Figure 4.

(a) Myeloid cell recovery occurred much faster than lymphoid cell recovery after irradiation and transplantation in mice. Percentage of CD19-positive B-cells and Mac-1-positive myeloid cells was measured in the peripheral blood 2 days before transplantation (-2 days) and 12 or 26 days after transplantation, respectively. While B-cells fraction in the blood is normally larger than myeloid cell fraction in healthy animals (left panel, day -2), the percentage of myeloid cells is increased 12 days after transplantation (middle panel). This led to an altered myeloid cell / lymphoid cell ratio and explains the differences in the blood cell composition between 4 weeks and 10 weeks after in transplantation in Figure 8 of the manuscript. For this experiment, untransduced LSK cells were used for transplantation. **(b)** Overview about the gating strategy to distinguish between Ly5.1 and Ly5.2 or GFP+ and GFP- cells in the blood and the bone marrow, respectively. All cells were stained with anti-CD45.1-pacific blue and anti-CD45.2-PE/Cy.7 antibodies. Additionally, Hoechst 33258 was added directly before flow cytometry measurement and detected in the same channel as pacific blue. Alive Ly5.2 cells were gated as CD45.2+, CD45.1-, and Hoechst 33258- (middle panel). Finally, we used a GFP negative control mouse to create the gate for GFP (upper right panel). Cells located to the right of this gate were analyzed as GFP+, the other cells as GFP- (lower right panel). **(c)** Percentage of Mac-1+/F4/80+ monocytes/macrophages in the peripheral blood at 4 weeks and 10 weeks after transplantation and the bone marrow 10 weeks after transplantation. We did not observe a constant and reproducible difference between miR-182 transduced and non-transduced animals. Notably, a slight but significant reduced percentage of monocytes in the GFP+ fraction of miR-182 transduced animals could be detected in the peripheral blood 10 weeks after transplantation. **(d)** Percentage of B220+ B-cells in the peripheral blood at 4 weeks and 10 weeks after transplantation and the bone marrow 10 weeks after transplantation. We did not observe a constant and reproducible difference between miR-182 transduced and non-transduced animals. Notably, a slight but significant increased percentage of B-cells in the GFP+ fraction of miR-182 transduced animals could be detected in the peripheral blood 10 weeks after transplantation. **(e)** Percentage of GFP+ cells 3 days after infection (indicated as “before BMT” in the figure) with either scramble coding (grey bars) or miR-182 coding (black bars) pseudoviral particles and over the time in the peripheral blood and the bone marrow. Data indicated a better transduction efficiency of the scramble control coding lentiviral vector compared to the miR-182 coding vector. (4 weeks: scramble n=9 and miR-182 n=8; 10 weeks: scramble n=8 and miR-182 n=6; p-values indicated above the bars, ns - not significant. P values were calculated using unpaired Student’s t-test).

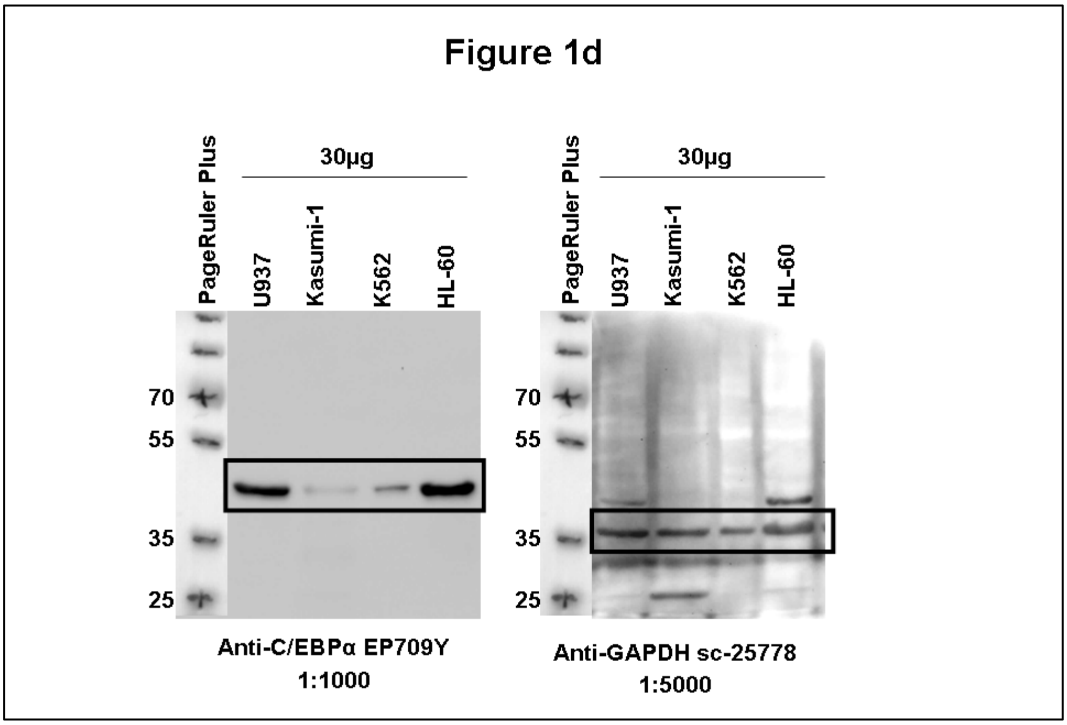

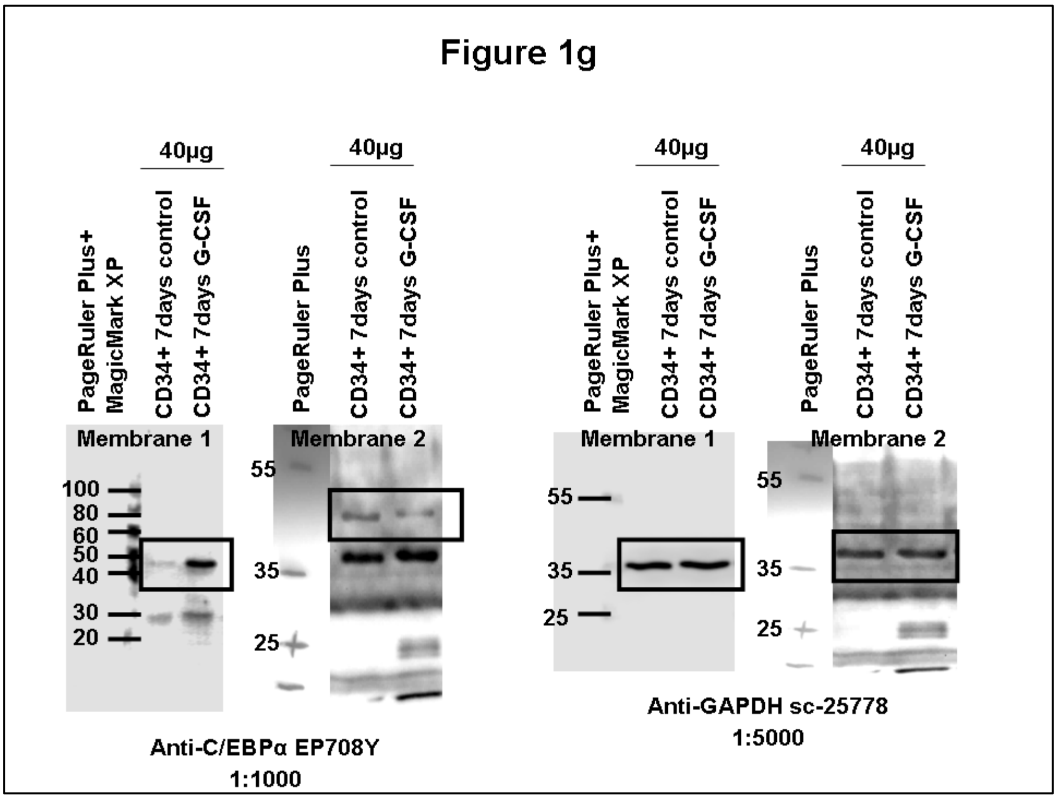

Supplementary Figure 5. Western Blot full gel scan for Figure 1

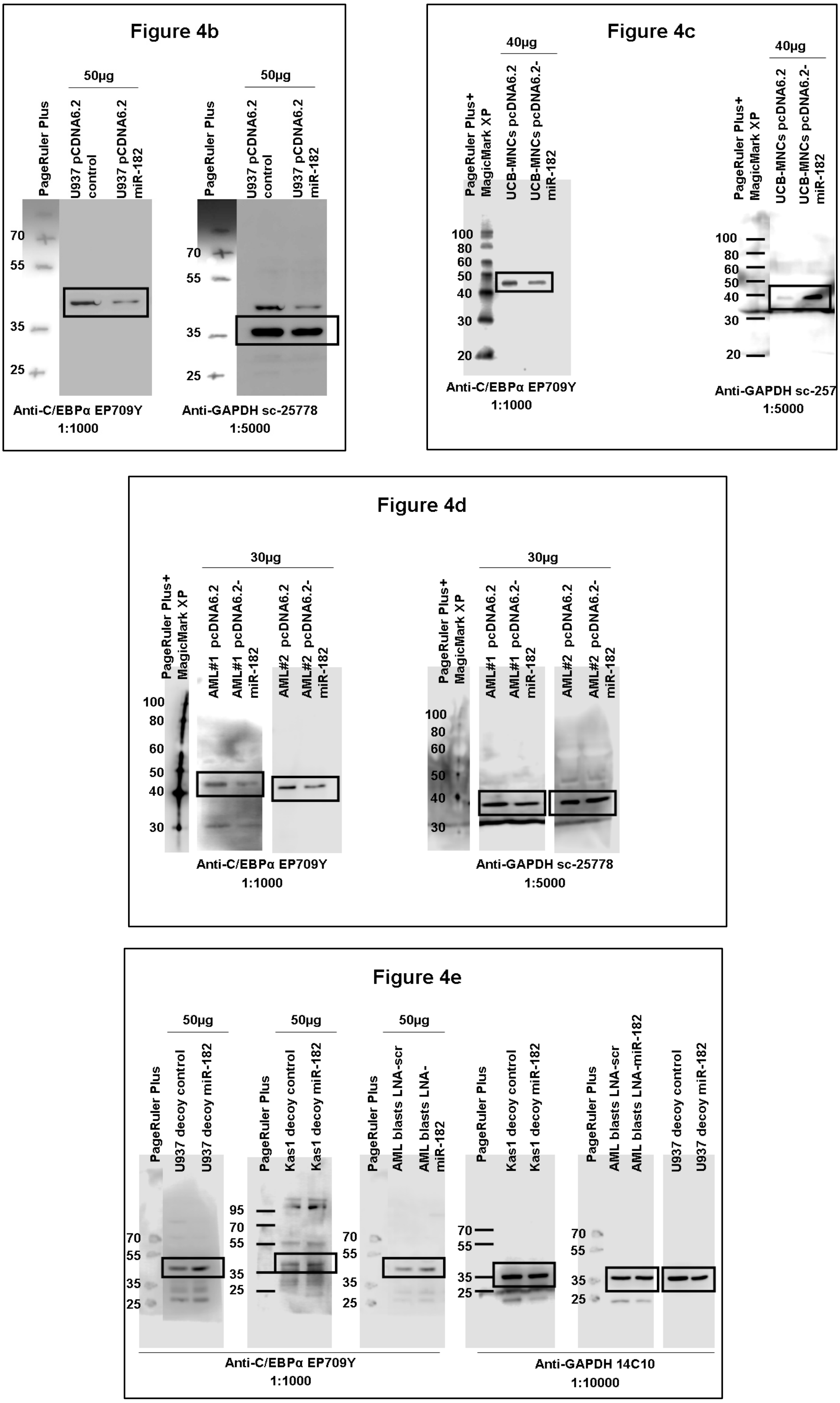

Supplementary Figure 6. Western Blot full gel scan for Figure 4

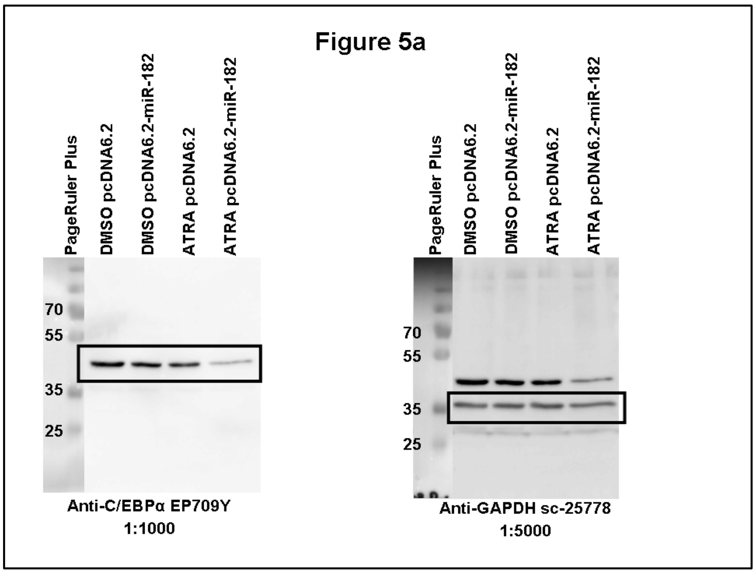

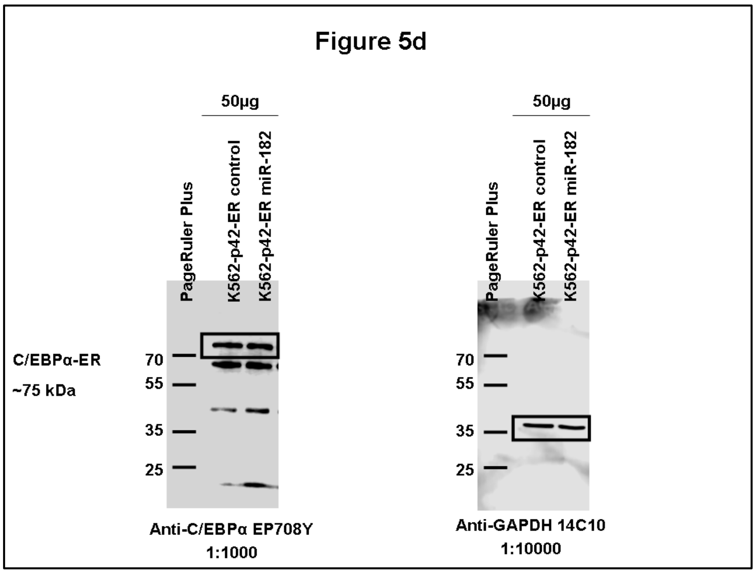

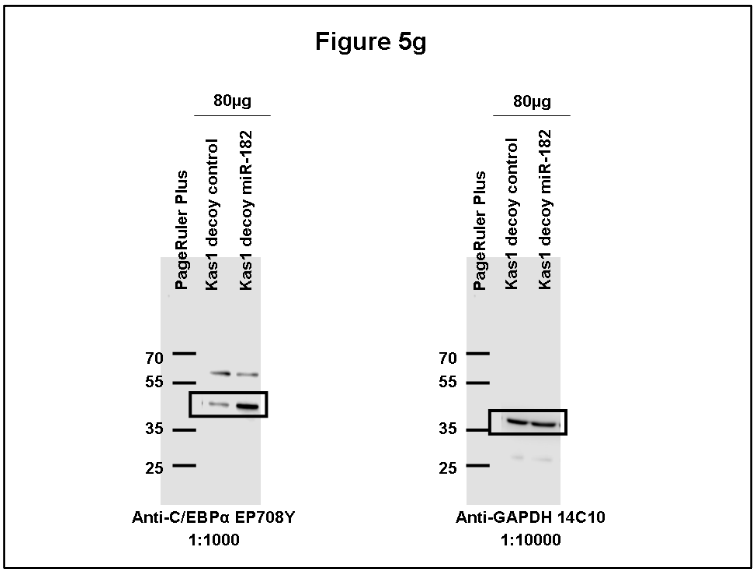

Supplementary Figure 7. Western Blot full gel scan for Figure 5

Figure 6g

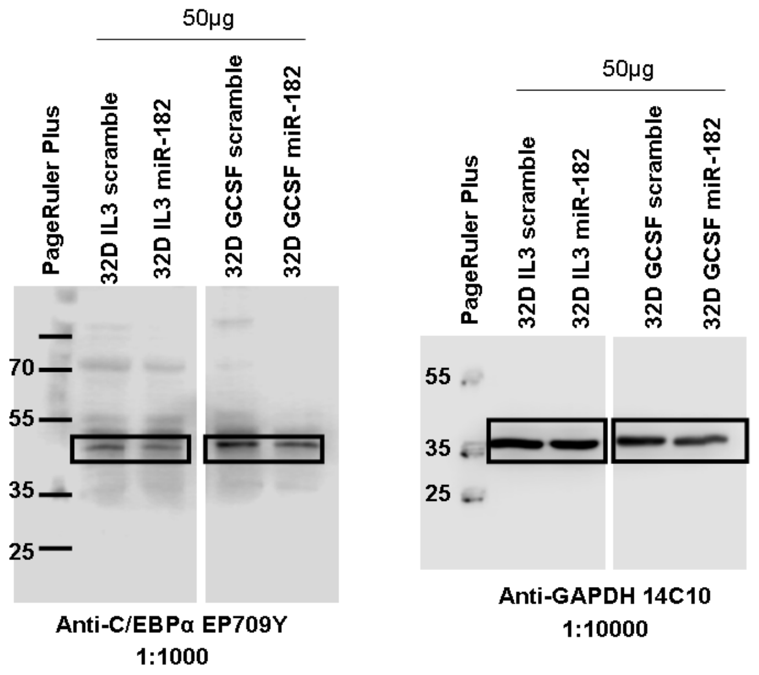

Figure 6h

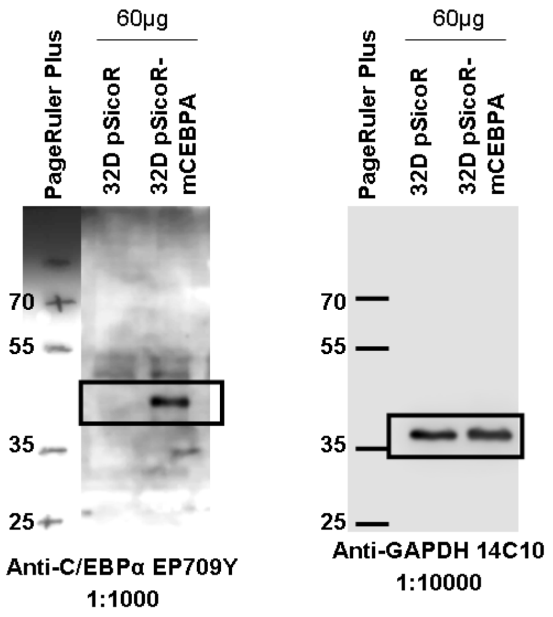

Supplementary Figure 8. Western Blot full gel scan for Figure 6

Supplementary Table 1.

List of microRNAs that are up-regulated by C/EBPα in K562-C/EBPα-ER cells. Cutoff for threshold mean was set at 1.5 fold. MicroRNAs in bold are known C/EBPα targets.

Human microRNA	Fold Change	SD
hsa-miR-34a-5p	***12.53***	***11.56***
hsa-miR-199b-3p	6.38	4.79
hsa-miR-29a-3p	***3.77***	***3.17***
hsa-miR-15b-3p	3.51	4.12
hsa-miR-143-3p	3.22	1.83
hsa-miR-221-3p	3.07	2.03
hsa-miR-23b-3p	2.79	1.30
hsa-miR-30c-5p	***2.77***	***2.91***
hsa-miR-299-5p	2.67	2.45
hsa-miR-770-5p	2.31	1.72
hsa-miR-212-5p	2.21	0.86
hsa-miR-454-5p	2.21	1.58
hsa-miR-24-3p	2.21	1.51
hsa-miR-641	2.16	1.81
hsa-miR-106b-5p	2.14	1.83
hsa-miR-625-3p	2.09	1.02
hsa-miR-223-3p	***2.05***	***1.33***
hsa-miR-145-5p	2.04	0.90
hsa-let-7d-5p	1.98	0.72
hsa-miR-431-5p	1.89	1.27
hsa-miR-361-3p	1.86	0.51
hsa-miR-766-3p	1.79	0.67
hsa-miR-181c-5p	1.70	0.95
hsa-miR-335-5p	1.63	0.89
hsa-miR-21-5p	1.56	0.27
hsa-miR-193b-3p	1.54	0.80
hsa-miR-615-3p	1.50	0.32
hsa-miR-3180-5p	1.50	0.64

Supplementary Table 2.

List of microRNAs that are down-regulated by C/EBPα in K562-C/EBPα-ER cells. Cutoff for threshold mean was set at 0.8 fold.

Human microRNA	Fold Change	SD
hsa-miR-3184-3p	0.80	0.12
hsa-miR-409-3p	0.80	0.07
hsa-miR-665	0.77	0.16
hsa-miR-1307-5p	0.76	0.05
hsa-miR-485-5p	0.75	0.10
hsa-miR-769-5p	0.75	0.09
**hsa-miR-182-5p**	**0.75**	**0.21**
hsa-miR-432-5p	0.72	0.26
hsa-miR-1185-2-3p	0.71	0.17
hsa-miR-25-5p	0.71	0.06
hsa-miR-4746-5p	0.71	0.02
hsa-miR-486-5p	0.70	0.22
hsa-miR-299-3p	0.69	0.15
hsa-miR-377-5p	0.65	0.20
hsa-miR-30e-3p	0.64	0.16
hsa-miR-27a-5p	0.64	0.10
hsa-miR-539-3p	0.64	0.28
hsa-miR-99b-3p	0.52	0.31
hsa-miR-92a-1-5p	0.51	0.20

Supplementary Table 3.

AML patient characteristics used for miR-182 expression analysis.

Patient no.	Age	Gender	Karyotype	*CEBPA* TAD1	*CEBPA* TAD2	*CEBPA* bZIP
**1**	36	F	healthy	-	-	-
**2**	41	M	healthy	-	-	-
**3**	41	M	healthy	-	-	-
**4**	77	F	normal	WT	WT	WT
**5**	82	F	normal	WT	WT	WT
**6**	73	M	normal	WT	WT	WT
**7**	64	M	normal	WT	WT	WT
**8**	67	F	normal	WT	WT	WT
**9**	73	F	normal	WT	WT	WT
**10**	70	F	normal	WT	WT	WT
**11**	39	F	normal	WT	WT	WT
**12**	44	F	normal	WT	WT	WT
**13**	59	M	normal	WT	WT	WT
**14**	78	F	normal	WT	WT	WT
**15**	48	M	normal	WT	WT	WT
**16**	50	M	normal	WT	***MUT***	WT
**17**	73	F	normal	***MUT***	WT	WT
**18**	60	F	normal	WT	***MUT***	WT
**19**	49	M	normal	WT	***MUT***	WT
**20**	60	F	normal	WT	***MUT***	WT
**21**	68	F	normal	MUT	WT	***MUT***
**22**	67	F	normal	MUT	WT	***MUT***
**23**	83	M	normal	MUT	WT	***MUT***
**24**	65	M	normal	WT	WT	***MUT***
**25**	66	F	normal	MUT	WT	***MUT***
**26**	56	M	normal	WT	WT	***MUT***
**27**	34	M	normal	WT	WT	***MUT***
**28**	77	F	normal	MUT	WT	***MUT***
**29**	47	M	normal	MUT	WT	***MUT***

Supplementary Methods

Supplementary Table 4 - Oligo list

	Primer Sequence
**expression analysis**	
mouse *CEBPA* forward	5’-GACCATTAGCCTTGTGTGTACTGTATG-3
mouse *CEBPA* reverse	5’-TGGATCGATTGTGCTTCAAGTT-3
GAPDH forward	5’-ACCACAGTCCATGCCATCAC-3’
GAPDH reverse	5’-TCCACCACCCTGTTGCTGTA-3’
human E2F1 forward	5’-ATGTTTTCCTGTGCCCTGAG-3’
human E2F1 reverse	5’-ATCTGTGGTGAGGGATGAGG-3’
**chromatin immunoprecipitation**	
#1 forward	5’-CACCTTGCTTGGCCTGTCT-3’
#1 reverse	5’-GATCAATGGTGCCGGATCGC-3’
#2 forward	5’-GTGCTGCCCTTGGGTAAGT-3’
#2 reverse	5’-CAGGAATGGGCCAAGCTAAAAC-3’
#3 forward	5’-CCTAGGGATGGTGTCTGCTCC-3’
#3 reverse	5’-TCGGTCTGTGCTGAGGAAGAG-3’
**miR-183-96-182 cluster analysis**	
F1	5‘-AGGGCCATAAACAGAGCAGAG-3‘
F2	5‘-TTGCTTGTGTCTCTCCGCTC -3‘
F3	5‘-AGTGCTGCTGATGTCCCAAA-3‘
R1	5‘-AGTGTGTGGTGTGTGAAGGG-3‘
R2	5‘-CACACTTCCGTGTTCCCACT -3‘
R3	5‘-CTACCATTGCCAAAAACGGGG -3‘
**gel shift assay**	
Site 1 forward	Biotin - 5‘-TGCAGGCGATCAATGGTGCCGGAT-3‘
Site 1 reverse	Biotin - 5‘-ATCCGGCACCATTGATCGCCTGCA -3‘
Site 3 forward	Biotin - 5‘-GCAACTGCGACAAGGTCCTTCCTG-3‘
Site 3 reverse	Biotin - 5‘-CAGGAAGGACCTTGTCGCAGTTGC-3‘
**promoter luciferase assay**	
Construct1-XhoI-for	5’-CTCGAGGTCCAGTGTCAGGCAGGAACGCTGTCCCAC-3’
Construct2-XhoI-for	5’- CTCGAGGCATGGGGATGCAGGGTTGGGGGCA-3’
MIR182-promoter-HindIII-rev	5’-AAGCTTCCCCCAGGAGCTGGGGAGGCCTCGGTCTGT-3’
C/EBP site 3 mut forward	5'-GGCGCCCATCCCCGCAACGCCGAGGGAGTCCTTCCTGCAGGGTCG-3'
C/EBP site 3 mut reverse	5’-CGACCCTGCAGGAAGGACTCCCTCGGCGTTGCGGGGATGGGCGCC-3’
E2F site 2 mut forward	5’-GCCTCTTCCCTAAAAGCAGTGGACAAACGCTAAAATTGGGACCTTCTACCCAGGAACGGGAACA-3’
E2F site 2 mut reverse	5’-TGTTCCCGTTCCTGGGTAGAAGGTCCCAATTTTAGCGTTTGTCCACTGCTTTTAGGGAAGAGGC-3’

Supplementary Table 5 - Flow cytometry antibody list

Antigen	Conjugate	Species	Company	Dilution
**cell lines**				
CD11b / Mac1	PE	human	BD Biosciences	1:50
CD114 / G-CSFR	APC	human	Biolegend	1:50
CD11b / Mac1	PE	mouse	eBioscience	1:50
**primary mouse cells**				
CD45.1	Pacific Blue	mouse	Biolegend	1:50
CD45.2	PE/Cy7	mouse	Biolegend	1:50
Ly-6G / Ly6C (Gr-1)	PerCP/Cy5.5	mouse	Biolegend	1:50
CD11b / Mac1	APC	mouse / human	Biolegend	1:50
CD4	PerCP/Cy5.5	mouse	Biolegend	1:50
CD45R / B220	APC	mouse / human	Biolegend	1:50
Ly-6A/E / Sca-1	APC	mouse	Biolegend	1:50
CD117 / c-Kit	PE	mouse	Biolegend	1:50
F4/80	PE	mouse	Biolegend	1:50
CD19	APC/Cy7	mouse	BD Biosciences	1:50
CD34	FITC	mouse	Biolegend	1:50
CD16/32 (FcgRII/III)	PE	mouse	Biolegend	1:50
Lineage Cocktail	Biotin	mouse	Miltenyi	1:10

Supplementary References

1. D'Alo, F. et al. The amino terminal and E2F interaction domains are critical for C/EBP alpha-mediated induction of granulopoietic development of hematopoietic cells. *Blood* **102**, 3163-71 (2003).
2. TCGA. Genomic and epigenomic landscapes of adult de novo acute myeloid leukemia. *N Engl J Med* **368**, 2059-74 (2013).
